# Label-free profiling of DNA aptamer-small molecule binding using T5 exonuclease

**DOI:** 10.1093/nar/gkaa849

**Published:** 2020-10-14

**Authors:** Obtin Alkhamis, Weijuan Yang, Rifat Farhana, Haixiang Yu, Yi Xiao

**Affiliations:** Department of Chemistry and Biochemistry, Florida International University, 11200 SW 8th Street, Miami, FL 33199, USA; Department of Chemistry and Biochemistry, Florida International University, 11200 SW 8th Street, Miami, FL 33199, USA; Department of Chemistry and Biochemistry, Florida International University, 11200 SW 8th Street, Miami, FL 33199, USA; Department of Chemistry and Biochemistry, Florida International University, 11200 SW 8th Street, Miami, FL 33199, USA; Department of Chemistry and Biochemistry, Florida International University, 11200 SW 8th Street, Miami, FL 33199, USA

## Abstract

*In vitro* aptamer isolation methods can yield hundreds of potential candidates, but selecting the optimal aptamer for a given application is challenging and laborious. Existing aptamer characterization methods either entail low-throughput analysis with sophisticated instrumentation, or offer the potential for higher throughput at the cost of providing a relatively increased risk of false-positive or -negative results. Here, we describe a novel method for accurately and sensitively evaluating the binding between DNA aptamers and small-molecule ligands in a high-throughput format without any aptamer engineering or labeling requirements. This approach is based on our new finding that ligand binding inhibits aptamer digestion by T5 exonuclease, where the extent of this inhibition correlates closely with the strength of aptamer-ligand binding. Our assay enables accurate and efficient screening of the ligand-binding profiles of individual aptamers, as well as the identification of the best target binders from a batch of aptamer candidates, independent of the ligands in question or the aptamer sequence and structure. We demonstrate the general applicability of this assay with a total of 106 aptamer-ligand pairs and validate these results with a gold-standard method. We expect that our assay can be readily expanded to characterize small-molecule-binding aptamers in an automated, high-throughput fashion.

## INTRODUCTION

Aptamers are short nucleic acids that bind to specific molecules with high affinity. They are isolated from random oligonucleotide libraries through an *in vitro* process known as systematic evolution of ligands by exponential enrichment (SELEX) to bind a variety of targets ranging from individual ions to whole cells (1–3). Aptamers have gained considerable attention as artificial bioreceptors for bioanalytical and therapeutic applications, as they offer several advantages relative to antibodies, such as high chemical stability, low batch-to-batch variation, and economical synthesis (3,4). Due to these and other advantageous properties, there has been increasing interest in the use of aptamers as probes for detecting small molecules relevant for biomedical research applications, medical diagnostics, therapeutic drug monitoring and drug testing (3,5), as well as the in-depth study of biological systems such as neurotransmission (6) and gene expression (7). For example, aptamers were recently employed for real-time monitoring of the pharmacokinetics of small-molecule drugs in the circulation of live animals (8). This is an especially promising application that could show clinical potential in the near future, which is likely considering that aptamers have already been approved as therapeutics (e.g. pegaptanib for macular degeneration) and several are currently in clinical trials as treatments for diseases such as cancer (9).

To be of practical use, aptamers need to have an appropriate level of affinity and specificity to a given set of ligands. For example, accurate diagnostic detection of disease-related analytes or biomarkers in biological specimens requires aptamers that bind strongly to a single target without any cross-reactivity to the myriad of interferents commonly present in complex biological matrices. On the other hand, applications that require the detection of large families of structurally-related compounds such as antibiotics (10) or illicit drugs (11) require aptamers with high affinity and broad cross-reactivity to the target family, but with tightly controlled specificity against those outside that family of compounds. However, finding aptamers with satisfactory binding profiles for these various applications is a challenging task. After several rounds of SELEX, tens to hundreds of aptamer candidates (11–13) can be identified through DNA sequencing methods such as Sanger sequencing or high-throughput sequencing on the basis of their prevalence or degree of enrichment (14). However, the binding properties of these candidates is not readily apparent, and a thorough comprehensive characterization of the affinity of each candidate sequence towards the target(s) and relevant interferents is the only means of identifying suitable aptamers. Existing affinity characterization methods that rely on specialized instrumentation such as isothermal titration calorimetry (ITC) (15), surface plasmon resonance (16), and microscale thermophoresis (17) can measure in-depth quantitative binding parameters such as binding affinity, enthalpy, entropy, stoichiometry, as well as on- and off-rate constants. However, these methods can only be used to study a single aptamer-ligand pair at a time, and are thus impractical for screening hundreds of candidates.

Simpler competition-based assays offer greater throughput while providing pertinent—but sometimes limited—thermodynamic information. One robust method is the strand-displacement assay, which was first developed by Hu and Easly (18) and later modified by Stojanovic and coworkers into a fluorescence microplate format (19). This involves the ligand-induced displacement of a complementary DNA strand that is hybridized to an aptamer, where the extent of displacement can be monitored by labeling the oligonucleotides with fluorophore–quencher pairs. The resulting binding curves can be used to ascertain aptamer target-binding affinity and specificity. However, this requires the use of chemically-labeled nucleic acids, which makes the screening of more than a few aptamer–ligand pairs highly impractical. Alternatively, dye-displacement assays offer a label-free approach. Certain small-molecule-binding aptamers have the capability of binding dyes such as thiazole orange (20,21), SYBR Green I (22), and diethylthiacarbocyanine (also known as Cy7) (11,23,24). In some cases, the binding of a ligand to aptamer-dye complexes can induce displacement of the dye, which results in a concomitant change in the fluorescence or absorbance of the dye that can in turn be used to assess aptamer binding. These methods are not universally applicable, however, because not all aptamers display the ability to bind and release dyes in a ligand-binding-dependent manner (20). Gold nanoparticle-based assays offer a label-free and more generalizable alternative for preliminary assessment of aptamer-ligand binding based on a colorimetric readout. Aptamers can adsorb onto gold nanoparticles, which prevents the nanoparticles from aggregating upon the addition of salt. When a ligand binds to the aptamer, the aptamer is released from the particle surface, and the addition of salt results in nanoparticle aggregation and a color change (25,26). Nonetheless, this method is prone to false positives or negatives due to the non-specific aggregation of gold nanoparticles as a result of factors such as buffer components, aptamers with complex structures, and even certain ligands themselves (27–29). Thus, there is a paucity of facile, scalable, and broadly applicable approaches for studying aptamer-ligand interactions in a high-throughput manner.

Here, we developed a novel high-throughput, label-free approach to profile the binding and interactions between DNA aptamers and small molecules in solution using T5 exonuclease (T5 Exo). This enzyme has 5′-3′ exonuclease activity on both single- and double-stranded DNA as well as single-strand endonuclease and 5′-flap endonuclease activity (30–32). T5 Exo has been widely used in the Gibson Assembly method for connecting fragments of DNA (33). However, no study has reported on the interaction between T5 Exo and ligand-bound DNA substrates. We for the first time discovered that the binding of small molecules to DNA aptamers inhibits their digestion by T5 Exo, and we used this enzyme to probe the binding of ligands to aptamers. We determined that the strength of aptamer-ligand binding is proportional to the enzymatic digestion rate and the aptamer's resistance to digestion, which enables the comparison of an aptamer's affinity for different ligands and therefore the evaluation of aptamer specificity. We exploited this phenomenon to develop a T5 Exo-based fluorescence assay for thoroughly profiling aptamer binding in a high-throughput microplate format. This assay distinguishes compounds that can or cannot bind to a particular aptamer with a degree of sensitivity that enables comparison of ligand-binding strengths among structurally related molecules or interferents that could be present in the intended sample matrix. In addition, we demonstrated that this assay can be used to screen among different aptamers for their ability to bind a particular ligand. We have demonstrated the widespread utility of our assay with six different aptamer–ligand systems, accounting for an overall total of 79 ligands and 33 aptamers. The accuracy of our method is confirmed by the gold standard characterization technique ITC or by previous reports. Advantageously, our method does not require aptamer labeling or engineering, prior knowledge of the target binding domain, and has no influence from aptamer sequence and tertiary structure. This is highly valuable for screening batches of aptamer candidates for their suitability in a variety of real-world applications. We envision that with a liquid-handling system, this method can be expanded to accurately profile hundreds or thousands of DNA aptamer-small-molecule ligand pairs in an automated fashion, greatly expediting the aptamer characterization process.

## MATERIALS AND METHODS

### Oligonucleotides

All DNA oligonucleotides were purchased from Integrated DNA Technologies with HPLC purification. Oligonucleotides were dissolved in PCR-grade water and DNA concentrations were measured with a NanoDrop 2000 Spectrometer. The sequences of the oligonucleotides used in this work are listed in the Supplementary Information (Supplementary Table S1).

### Experimental conditions

Enzyme digestion experiments were performed at 25°C. SELEX was performed at room temperature (∼20°C). Isothermal titration calorimetry (ITC) experiments were performed at 23°C. Experiments with each aptamer utilized the following reaction buffers: ATP aptamers (10 mM Tris–HCl, pH 7.4, 10 mM MgCl_2_), MA aptamers (10 mM Tris–HCl, pH 7.4, 20 mM NaCl, 5 mM MgCl_2_), MMC aptamers (10 mM Tris–HCl, pH 7.4, 20 mM NaCl, 0.5 mM MgCl_2_), SCA2.1 aptamer (10 mM Tris–HCl, pH 7.4, 20 mM NaCl, 0.5 mM MgCl_2_), and dopamine aptamer (10 mM phosphate buffer, pH 7.4, 140 mM NaCl, 4 mM KCl, 2 mM MgCl_2_). For experiments involving exonucleases, 0.1 mg/ml bovine serum albumin was included in the reaction buffer. For aptamer isolation, the following buffer was used: 10 mM Tris–HCl, pH 7.4, 20 mM NaCl, 0.5 mM MgCl_2_.

### Exonuclease digestion assays and gel electrophoresis analysis

For all digestion assays, 1 μl of 50 μM aptamer was mixed with 44 μl reaction buffer containing the appropriate concentration of target. After incubation for one hour, 5 μl of 2 U/μl T5 Exo or 5 μl of a mixture of 2 U/μl T5 Exo and 0.15 U/μl Exo I was added to the solution. A 5 μl quantity of the reaction mixture was collected at various time points and mixed with 15 μl of formamide loading buffer (75% formamide, 10% glycerol, 0.125% SDS, 10 mM EDTA, and 0.15% (w/v) xylene cyanol) to quench the reaction. Digestion products were then analyzed by 15% denaturing polyacrylamide gel electrophoresis (PAGE). Separation was carried out at 20 V/cm for 2.5–3.5 h in 0.5× TBE buffer. The gel was stained with 1× SYBR Gold for 25 min and imaged using a ChemiDoc MP Image system (BioRad).

### Exonuclease-based profiling fluorescence microplate assays

A 1 μl quantity of 50 μM MA-46 or 50 μM MMC1, 25 μM SCA2.1, or 25 μM dopamine aptamer was mixed with 44 μl of their respective reaction buffer containing an appropriate concentration of ligand. For the MA-46 calibration curve, 0, 25, 50, 100, 200, 400 and 800 μM MDPV was used (final concentration). For aptamer-ligand profiling experiments, 400, 200, 50 or 200 μM ligand was used for MA-46, MMC1, SCA2.1, or dopamine aptamer respectively (final concentration); ligand-free controls were included as well. For screening of MMC aptamers binding mephedrone, 0 or 200 μM mephedrone was used (final concentration). The aptamer and ligand were incubated for 1 h. Then, 5 μl of a mixture containing 2 U/μl T5 Exo and 0.15 U/μl Exo I was added to the solution. A time-course of fluorescence was recorded for 1.5 h for MA-46, 4 h for MMC1, 3 h for SCA2.1 or 2.5 h for dopamine aptamer by mixing 5 μl of the reaction mixture collected at different time points with 25 μl of a quenching solution (1.2× SYBR Gold, 12 mM Tris–HCl (pH 7.4), 3.75 mM EDTA, and 48% (v/v) formamide) pre-loaded in the wells of a black 384-well microplate. Fluorescence emission spectra from 500 to 800 nm and emission at 545 nm were acquired using a Tecan M1000 Pro microplate reader with 495 nm excitation. An aptamer's resistance to digestion (resistance value) was quantified by using the equation (AUC_1_ – AUC_0_)/AUC_0_ where AUC_1_ and AUC_0_ are the areas under the curve of the fluorescence time course plots with and without ligand, respectively. Cross-reactivity was calculated using the equation (AUC_L_ – AUC_0_)/(AUC_T_ – AUC_0_) × 100, where AUC_L_ and AUC_T_ is the resistance of aptamer digestion in the presence of a given ligand and the aptamer's main target (MDPV for MA-46, mephedrone for MMC1, MDPV for SCA2.1, and dopamine for the dopamine-binding aptamer), respectively.

### Cross-reactivity determination via strand-displacement fluorescence assay

First, to optimize the concentration of the complementary DNA strand to quench aptamer fluorescence by >90%, 40 μl of various concentrations (final concentrations: 0, 12.5, 25, 50, 100, 200, 400 nM) of a 15-nt complementary DNA strand labeled with 3′-dabcyl (termed dab-15) was incubated with 40 μl 5′ fluorescein-labeled MA-46 (MA-FAM) in reaction buffer at 95°C for 5 min. Thereafter, the solution was cooled over 30 min to room temperature. A 75 μl quantity of this solution was loaded into the wells of a black 384-well microplate. Fluorescence emission spectra from 510 to 800 nm were recorded with excitation at 495 nm. Under the optimized conditions, a 75 μl solution containing MA-FAM and dab-15 (final concentrations 50 and 100 nM, respectively) dissolved in reaction buffer was incubated at 95°C for 5 min and then cooled down to room temperature over 30 min. Then, 5 μl of ligand (final concentration: 400 μM) was added to the solution and incubated for 30 min. A ligand-free solution was prepared as a control. Afterwards, 75 μl of the solution was loaded into the wells of a black 384-well microplate. The fluorescence emission spectra were recorded from 510 to 800 nm with 495 nm excitation. Signal gain was calculated using the equation (*F* − *F*_0_)/*F*_0_, where *F* and *F*_0_ represent fluorescence intensity in the presence and absence of ligand, respectively. Cross-reactivity was calculated using the equation (*S*_L_/*S*_T_) × 100, where *S*_T_ is the signal gain produced by the main target of the aptamer (MDPV) and *S*_L_ is the signal gain produced by a given ligand.

## RESULTS

### Ligand binding inhibits T5 Exo digestion of a stem-loop-structured ATP aptamer

**Figure 1.**
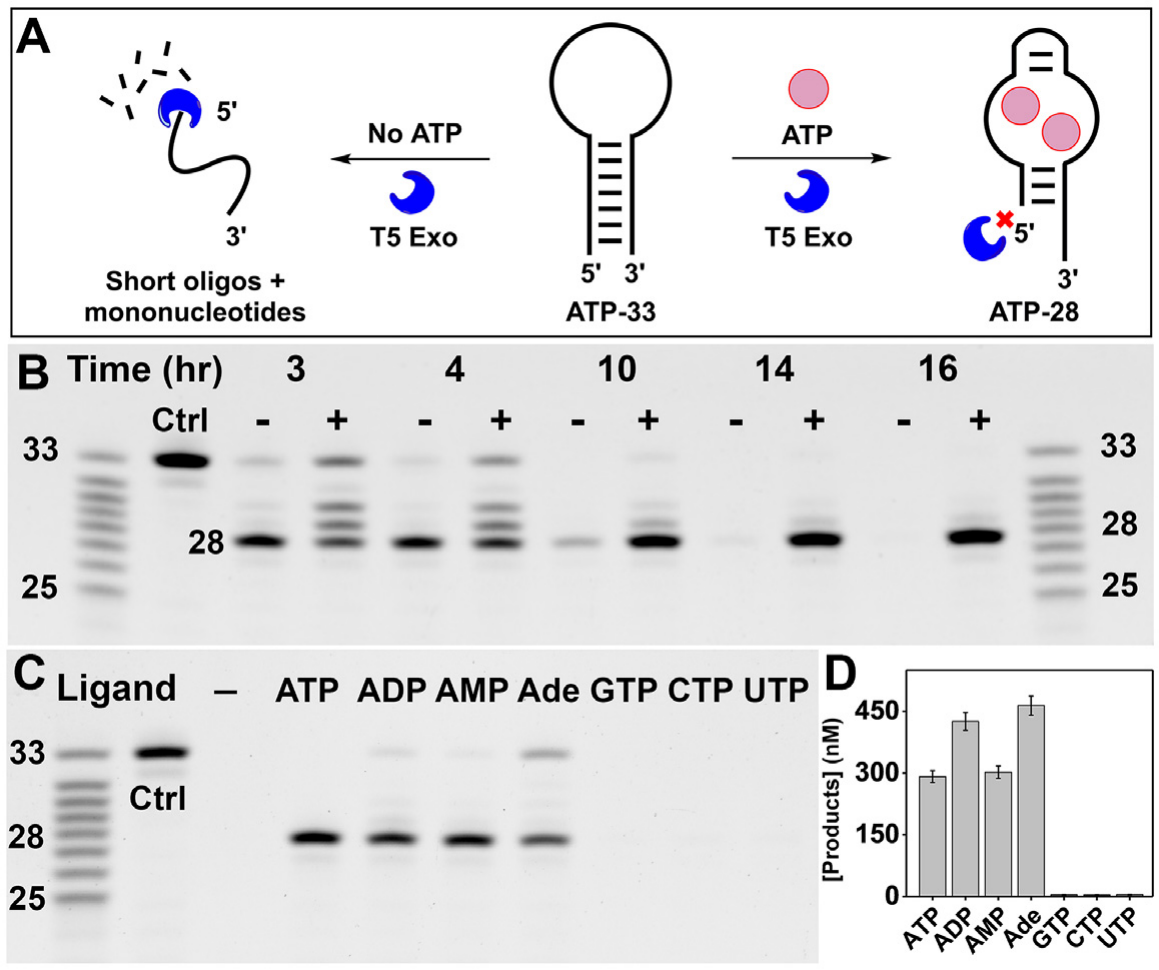
Binding-dependent digestion of ATP-binding aptamer ATP-33 by T5 Exo. (**A**) Scheme of T5 Exo digestion of ATP-33 in the absence (left) and presence (right) of ATP. (**B**) Time course digestion of ATP-33 by T5 Exo in the absence (−) or presence (+) of 250 μM ATP analyzed by polyacrylamide gel electrophoresis (PAGE). (**C**) Digestion of ATP-33 by T5 Exo in the absence or presence of 250 μM ATP, ADP, AMP, adenosine (Ade), GTP, CTP, or UTP after 16 h. (**D**) Total concentrations of the parent aptamer and 28-nt major product from the gel in panel C, as calculated relative to corresponding ladder bands. Error bars indicate the standard deviation of two experiments.

To assess whether aptamer–ligand binding inhibits aptamer digestion by T5 Exo, we used a well-characterized 33-nt DNA aptamer that binds to ATP (ATP-33) (34) derived from the aptamer isolated by Huizenga and Szostak (35). We digested ATP-33 with T5 Exo in the absence and presence of ATP, and analyzed the digestion process using polyacrylamide gel electrophoresis (PAGE). In the absence of target, the aptamer was exonucleolytically digested into a 28-nt major product that was eventually degraded in 14 h (Figure 1A, left). We also observed that T5 Exo exerted endonuclease activity based on the presence and accumulation of a shorter product generated discontinuously that migrated further below on the gel (Supplementary Figure S1). However, in the presence of ATP, the28-nt product persisted and accumulated over time due to its apparent resistance to digestion (Figure 1A, right and Figure 1B). This result indicated that ATP interferes with the ability of T5 Exo to digest this aptamer. We synthesized the resulting 28-nt digestion product, termed ‘ATP-28’ (Supplementary Table S1), and determined via ITC that it has similar micromolar ATP-binding affinity (*K*_d1_ = 4.1 ± 0.2 μM and *K*_d2_ = 14.3 ± 0.2 μM) (Supplementary Figure S2) to the parent aptamer (ATP-33) (34) (*K*_d1_ = 0.6 ± 0.1 μM and *K*_d2_ = 12.3 ± 0.6 μM). We posited that ATP binding to the aptamer, rather than ATP itself deactivating the enzyme, was directly responsible for this phenomenon. To test this, we digested a point mutant of ATP-33 (ATP-33-M; Supplementary Table S1) that has very weak affinity for ATP (*K*_d_ = 291 μM) (34) with T5 Exo. We found that the mutant was completely digested within two hours and the digestion profile was identical regardless of the presence or absence of ATP (Supplementary Figure S3). Together, these findings showed that ATP binding to ATP-33 directly inhibits digestion of the aptamer by T5 Exo.

The sensitivity of T5 Exo to the aptamer binding state could enable one to probe the extent to which an aptamer binds a given ligand. To demonstrate this, we digested ATP-33 with T5 Exo in the absence and presence of ATP or several related analogs including adenosine diphosphate (ADP), adenosine monophosphate (AMP), adenosine, and other nucleotide triphosphates including guanidine triphosphate (GTP), uridine triphosphate (UTP), and cytidine triphosphate (CTP). The aptamer was completely digested in the absence of any ligand, but inhibition of aptamer degradation was evident when the aptamer was digested in the presence of ATP, ADP, AMP and adenosine (Figure 1C). On the other hand, the aptamer was completely digested in the presence of GTP, UTP and CTP. This implies that ATP-33 can bind to all adenosine analogs but not nucleotide trisphosphates in general, which reflects the binding profile of this aptamer as reported by originally by Huizenga and Szostak (35). Moreover, aptamer digestion in the presence of each ligand resulted in a different amount of retained product and the level of enzymatic inhibition for each analog coincided with their previously reported cross-reactivity (17,36,37). For example, more 28-nt product (as well as undigested aptamer) was retained for ADP and adenosine relative to ATP and AMP (Figure 1D), which implies that the aptamer binds more strongly to the former pair. The reason for this could be that adenosine-bound ATP-33 has lower affinity for T5 Exo than the free aptamer and the aptamer bound to the other analogs (e.g. ATP, ADP, AMP). This may explain why ATP-33 itself is protected from digestion to a greater extent in the presence of adenosine. These results therefore indicate that T5 Exo can be used to sensitively profile the relative binding strength of an aptamer for various ligands in a facile manner.

### Demonstrating the generality of the T5 Exo assay with a three-way-junction structured aptamer

To determine whether digestion of aptamers by T5 Exo is generally inhibited by aptamer-target binding, we tested an aptamer recently isolated for the small-molecule drug 3,4-methylenedioxypyrovalerone (MDPV). This aptamer, termed MA-46 (Supplementary Table S1), is 46 nt in length, has a three-way-junction structured target-binding domain, and can bind several analogs of MDPV (24). We first digested MA-46 with T5 Exo in the absence and presence of MDPV. The aptamer was completely digested in the absence of MDPV within 2 h, but a 41-nt major product remained in the presence of the target. As with the digestion of ATP-33, we observed both exonuclease and endonuclease digestion products for MA-46 (Supplementary Figure S4). We synthesized this major product, MA-41 (Supplementary Table S1), and confirmed via ITC that it binds MDPV (*K*_d_ = 24.8 ± 0.7 μM) with similar affinity to the parent aptamer (MA-46) (*K*_d_ = 18.8 ± 1.4 μM) (Supplementary Figure S5).

**Figure 2.**
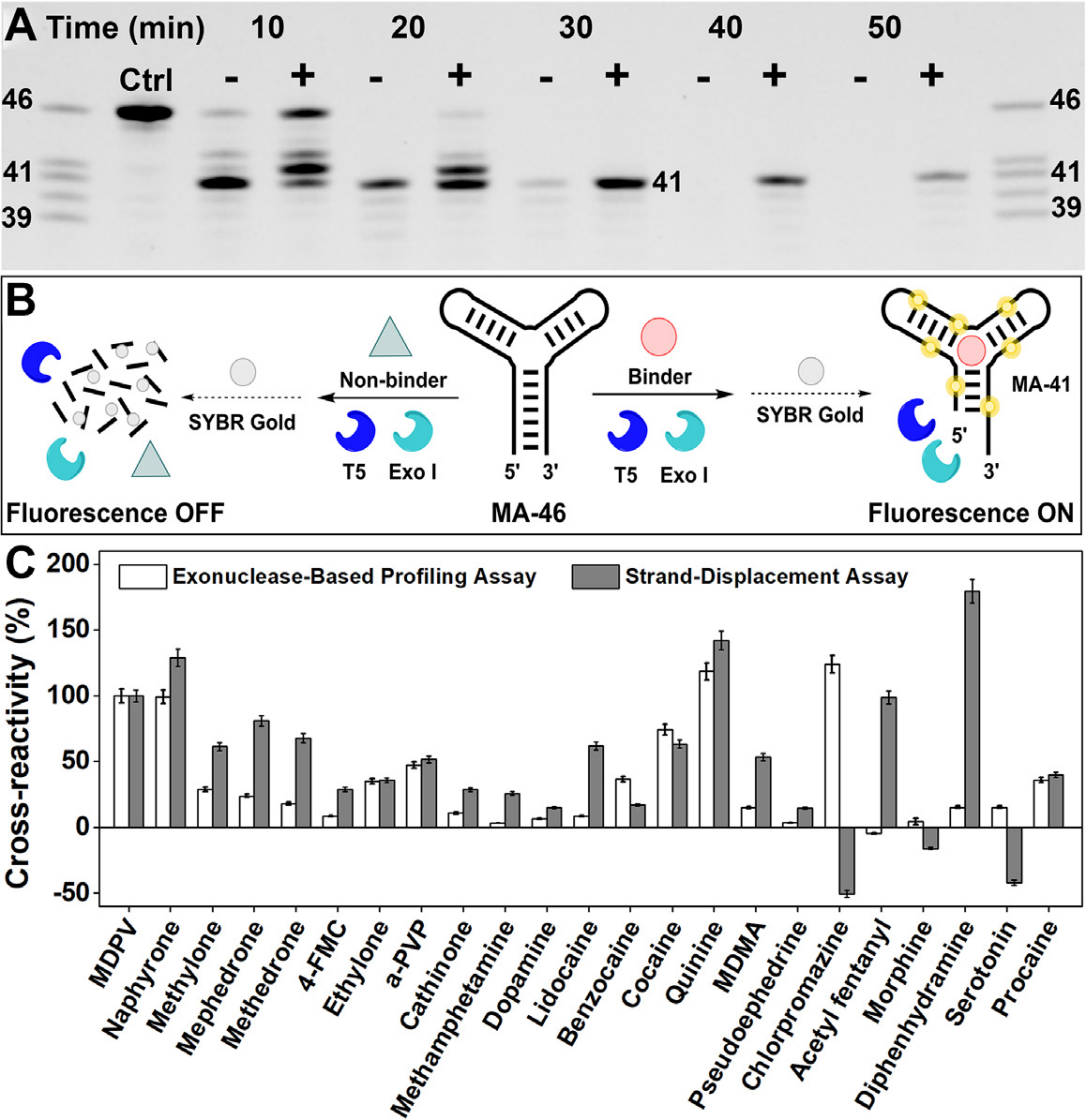
Profiling ligand binding of an MDPV-binding aptamer (MA-46) with an exonuclease mixture. (**A**) PAGE analysis of time-course digestion of MA-46 by a mixture of T5 Exo and Exo I in the absence (−) or presence (+) of 250 μM MDPV. (**B**) Scheme of the exonuclease-based ligand-profiling fluorescence assay. (**C**) Cross-reactivities of MA-46 to 29 different ligands in the exonuclease-based profiling assay (white bars) and a strand-displacement assay (gray bars), where cross-reactivity is calculated relative to the signal produced by MDPV. Error bars indicate the standard deviation of three experiments.

As a means for accelerating aptamer digestion, we next digested MA-46 with a mixture of T5 Exo and exonuclease I (Exo I), an enzyme that rapidly digests single-stranded DNA in the 3′-to-5′ direction (38). We hypothesized that the addition of Exo I would aid in the removal of single-stranded products generated by T5 Exo, thereby increasing the overall rate of aptamer digestion. We obtained a similar digestion profile with this exonuclease mixture compared to T5 Exo alone, although the time needed to completely digest the aptamer in the absence of target reduced by 3-fold to 40 min (Figure 2A). To verify that formation of the target-aptamer complex is directly responsible for enzymatic inhibition, we designed a point-mutant of MA-46 (MA-Mutant) (Supplementary Table S1) in which we substituted the thymine at position 9 with guanine, and confirmed using ITC that the mutant has no affinity for MDPV (Supplementary Figure S6). The digestion profile and time required to complete digestion of the mutant in this enzyme mixture was the same regardless of the absence or presence of MDPV (Supplementary Figure S7). In contrast, with the original MA-46 aptamer, we observed a target concentration-dependent increase in the retention of the 41-nt product with increasing concentrations of MDPV (0 to 800 μM) (Supplementary Figure S8). These results indicated that MDPV binding to the aptamer is directly responsible for the inhibition of aptamer digestion.

### A high-throughput microplate-based exonuclease assay for aptamer-ligand profiling

Having determined that the digestion of aptamers by T5 Exo and Exo I is sensitive to the binding state of an aptamer, we developed a label-free fluorescence microplate assay amenable for convenient, high-throughput profiling of aptamer-ligand binding interactions. If an aptamer binds a ligand, the aptamer will be largely spared from digestion, and the resulting truncated oligonucleotide products can be stained by the DNA-binding dye SYBR Gold (39), producing strong fluorescence that can be measured with a plate reader (Figure 2B, right). Non-binding ligands will fail to protect the aptamer from being digested; the resulting short oligonucleotides and mononucleotides cannot efficiently bind to the dye, which results in minimal fluorescence (Figure 2B, left). To demonstrate this concept, we first investigated if the amount of digestion product and the resulting fluorescence signal is proportional to the concentration of target. We digested MA-46 with T5 Exo and Exo I in the presence of varying concentrations of MDPV (0–800 μM) and quenched the reaction at 40 min with EDTA and formamide, followed by the addition of SYBR Gold. As expected, increasing concentrations of MDPV resulted in greater SYBR Gold fluorescence (Supplementary Figure S9) due to retention of a greater amount of undigested and slightly digested aptamers.

We next digested MA-46 in the presence of various ligands and recorded a time-course of fluorescence by quenching the reaction at different time intervals, followed by quantitative measurement of the digestion products with SYBR Gold. We expected that if MA-46 bound a ligand, it would exhibit a slower rate of digestion relative to samples without ligands or with ligands that fail to bind, where the aptamers would be digested at the normal rate. We tested 24 compounds, including nine members of the synthetic cathinone family (MDPV, naphyrone, methylone, mephedrone, methedrone, 4-fluoromethcathinone (4-FMC), ethylone, α-pyrrolidinopentiophenone (alpha-PVP) and cathinone), four non-cathinone compounds that are structurally related to MDPV (methamphetamine, dopamine, methylenedioxymethamphetamine (MDMA), and pseudoephedrine), three structurally-diverse compounds (acetyl fentanyl, morphine, and serotonin) and eight ligands thought to generally bind three-way-junction structured aptamers (lidocaine, benzocaine, cocaine, quinine, chlorpromazine, diphenhydramine, and procaine) (see Supplementary Figure S10 for structures) (24,40). The digestion of the aptamer in the absence of ligand occurred at an exponential rate, with the lowest level of fluorescence attained within 1.5 h. Different digestion trends were observed with the various ligands. For example, digestion in the presence of ethylone resulted in slightly greater fluorescence over the whole-time course relative to the ligand-free sample, suggesting that ethylone binds to the aptamer, albeit weakly. Digestion of the aptamer in the presence of ligands with greater binding affinity, such as MDPV and quinine, resulted in higher levels of aptamer retention (Supplementary Figure S11). The area under the time-plot curves, which corresponds to the integral of fluorescence with respect to time, is proportional to an aptamer's susceptibility to enzymatic digestion. We defined the contribution of aptamer-ligand binding to enzymatic resistance by using the metric we term the ‘resistance value’, which correlates aptamer ligand-binding affinity with the kinetics of enzymatic digestion without any bias related to enzyme activity, aptamer sequence or sequence motifs, or the structure of the aptamers. The resistance value equates to the ratio of the difference between the area under the curves of aptamer digestion with and without ligand minus 1. The resulting metric allows for an accurate assessment of aptamer–ligand binding strength, where higher resistance values imply tighter ligand-aptamer binding and vice-versa. The exonuclease profiling method revealed that MA-46 cross-reacts with all synthetic cathinones as well as non-targets such as benzocaine, chlorpromazine, cocaine, and procaine (Figure 2C), which is corroborated by our previous findings with this aptamer (24,41). Using this method, we were also able to identify new ligands that bind to MA-46, such as MDMA and serotonin as well as non-binding ligands such as acetyl fentanyl and morphine, and these results were verified by ITC (Supplementary Figure S12). Thus, our exonuclease profiling method enables us to comprehensively determine the binding spectrum of aptamers for different ligands that have varying structures and levels of affinity.

To validate these results, we also assessed the cross-reactivity of MA-46 to the above-mentioned compounds with a strand-displacement fluorophore-quencher assay (19,42). We first optimized the ratio of quencher (dabcyl)-labeled complementary strand (dab-15) to fluorophore (fluorescein)-labeled MA-46 (MA-FAM) to achieve high quenching efficiency (Supplementary Table S1, Figure S13). Under these optimized conditions, we challenged the complex with a fixed concentration of each ligand. The two assays showed close agreement regarding the cross-reactivity of most ligands, although some results were completely divergent—for example, for methamphetamine, lidocaine, chlorpromazine, acetyl fentanyl, diphenhydramine, and serotonin (Figure 2C). In order to better understand these disparate outcomes, we used ITC to determine the binding affinity of these compounds to MA-46. These results supported the findings of the exonuclease profiling method over those of the strand-displacement assay. ITC confirmed that chlorpromazine and serotonin bound to MA-46 with a *K*_d_ of 0.6 ± 0.1 and 55 ± 2 μM, respectively (Supplementary Figure S12), whereas the strand-displacement assay reported that these ligands had no cross-reactivity for the aptamer. Likewise, ITC verified the exonuclease-based finding that methamphetamine, lidocaine, acetyl fentanyl, and diphenhydramine had no or very weak affinity for MA-46 (*K*_d_ > 1 mM, >1 mM, 185 ± 47 and 195 ± 5 μM, respectively) (Supplementary Figure S12), whereas the strand-displacement assay showed 25–150% cross-reactivity to these compounds relative to MDPV. We suspected that the ligands themselves may have been affecting the readout from the fluorophore employed in the strand-displacement assay. To confirm this, a control experiment was performed by incubating fluorescein-labeled MA-46 or unmodified MA-46 mixed with SYBR Gold with and without these ligands. The results indicated that the fluorescence of MA-FAM was attenuated by chlorpromazine (–90%) and serotonin (–35%) and enhanced by acetyl fentanyl (+30%), and diphenhydramine (+30%) (Supplementary Figure S14). However, only chlorpromazine had a significant effect on the fluorescence of SYBR Gold, attenuating its fluorescence by 40% (Supplementary Figure S14). We believe these ligands may also affect the efficiency of the dabcyl quencher, since the effect on the fluorophore does not completely account for the relatively large false signal. Nevertheless, these findings delineate the robustness of the SYBR Gold readout used in the exonuclease profiling assay versus the fluorescein reporter used in the strand-displacement assay.

### Isolation of highly specific aptamers for the small-molecule drug mephedrone

As another demonstration of the exonuclease profiling method, we isolated and characterized new aptamers that have high specificity for the synthetic cathinone 4-methylmethcathinone (mephedrone). The SELEX procedure is detailed in Supplementary Table S2. Briefly, selection was performed for mephedrone with a stem-loop structured 73-nt DNA library containing a 30-nt random domain, representing ∼6 × 10^14^ unique oligonucleotides. The concentration of the target and library were gradually tapered down in each round to enrich high-affinity aptamers. We also employed a stringent counter-SELEX procedure (43) from the second round on to isolate aptamers that only bind to mephedrone but not structurally-similar molecules. The counter-selection regime included 21 synthetic cathinones, illicit drugs, and cutting agents/adulterants, and the concentration of these counter-targets was progressively increased throughout the selection process to eliminate cross-reactive aptamers. The progress of SELEX was monitored via the percent of target-specific pool elution for each round (Supplementary Figure S15A).

No significant target enrichment was observed during the first six rounds. After observing high cross-reactivity to counter-targets despite performing counter-SELEX, error-prone PCR was performed prior to the sixth round of selection to increase pool diversity and reduce the prevalence of cross-reactive sequences (43). Selection was subsequently performed for five more rounds without error-prone PCR. The percent of pool eluted by mephedrone began to increase by the seventh round and the pool demonstrated saturated binding to mephedrone after 11 rounds (Supplementary Figure S15A). Using a previously reported gel-elution assay (11), we determined that this pool bound to mephedrone with a *K*_d_ of 89 μM (Supplementary Figure S15B) and low to moderate binding to all counter-targets (Supplementary Figure S15C). Only the most structurally-similar compounds, such as methedrone, 4-FMC, methylone, ethylone, and cathinone showed notable pool elution relative to buffer. We cloned and sequenced the round 11 pool and obtained the sequence of 49 clones, of which 29 were unique (Supplementary Table S3). The most abundant sequence, termed MMC1 (Supplementary Table S1), encompassed 30% of the clones, and only four other sequences had more than two copies. Using ITC, we confirmed that MMC1 binds mephedrone with moderate affinity (*K*_d_ = 15 ± 1.3 μM) (Supplementary Figure S16).

### Exonuclease profiling of the ligand-binding spectrum of MMC1

We then studied the binding profile of MMC1 against mephedrone and the counter-targets using the exonuclease profiling fluorescence assay. We first digested MMC1 with T5 Exo with and without mephedrone and confirmed that the aptamer digestion is largely inhibited in the presence of target (Supplementary Figure S17, left). Unlike the digestion of ATP-33 and MA-46, MMC1 was continuously degraded nucleotide by nucleotide by T5 Exo (Supplementary Figure S17, right). This is most likely because the enzyme has greater exonuclease versus endonuclease activity at low Mg^2+^ concentrations (in this case, 0.5 mM Mg^2+^) (31). We then digested the aptamer with a mixture of T5 Exo and Exo I. The aptamer was completely digested in the absence of target, while a 42-nt major product persisted when the target was present (Supplementary Figure S18). We synthesized this digestion product as the oligonucleotide ‘MMC1–42’ (Supplementary Table S1) and found using ITC that it binds to mephedrone with slightly improved affinity (*K*_d_ = 6.6 ± 0.7 μM) relative to the parent aptamer (Supplementary Figure S19). To confirm that binding of mephedrone to the aptamer was directly responsible for enzymatic inhibition, we designed a point-mutant of MMC1 (MMC1-Mutant) by changing thymine at position 24 to cytosine and confirmed that the construct had no affinity for mephedrone (Supplementary Figure S20). We digested this mutant with the exonuclease mixture and observed similar digestion profiles regardless of the absence or presence of mephedrone (Supplementary Figure S21). With MMC1, we observed increased retention of the 42-nt product with increasing concentrations of mephedrone (0–800 μM) (Supplementary Figure S22). These results show that mephedrone binding to MMC1 directly impedes aptamer digestion.

**Figure 3.**
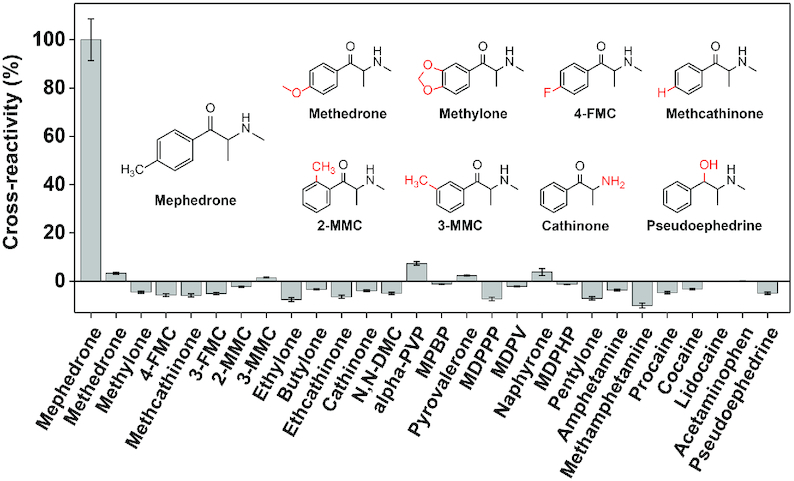
Assessing the specificity of mephedrone-binding aptamer MMC1 using the exonuclease-based profiling assay. Cross-reactivity of 20 synthetic cathinone analogs and seven non-cathinone targets, calculated relative to the aptamer resistance value for the sample containing mephedrone.

We then used the exonuclease fluorescence assay to determine the cross-reactivity of MMC1 towards mephedrone and 20 other synthetic cathinones as well as 7 non-cathinone compounds (Figure 3 and Supplementary Figure S23). The aptamer showed no significant cross-reactivity (< 10%) to all non-target compounds, including to methedrone, which differs from mephedrone by only a single oxygen atom. Importantly, MMC1 can also discriminate mephedrone from its positional isomers 2-MMC and 3-MMC. The aptamer is also generally highly sensitive to minor structural alterations in the target. For example, the replacement of the methyl group on the benzene with hydrogen in methcathinone or fluorine in 4-FMC severely impairs binding. Other alterations to the benzene ring (e.g. methylone), alkyl tail (e.g. pyrovalerone), or amino group (e.g. ethcathinone and cathinone) or removal of the ketone moiety (i.e. pseudoephedrine, amphetamine, methamphetamine) likewise impairs binding (Figure 3). These results show the plethora of detail that the exonuclease profiling method can provide on aptamer binding spectra. We also performed ITC to confirm the poor binding of MMC1 to methedrone, methylone, methcathinone, ethylone, 2-MMC, 3-MMC and 4-FMC. The binding affinity for methedrone was 10-fold lower than for mephedrone, and the other synthetic cathinones bound with at least 20–50-fold lower affinity or no affinity at all (Supplementary Figure S24). These results confirm that our exonuclease fluorescence assay can accurately profile aptamer binding in a high-throughput manner.

### Screening other mephedrone-binding aptamer candidates using the exonuclease assay

**Figure 4.**
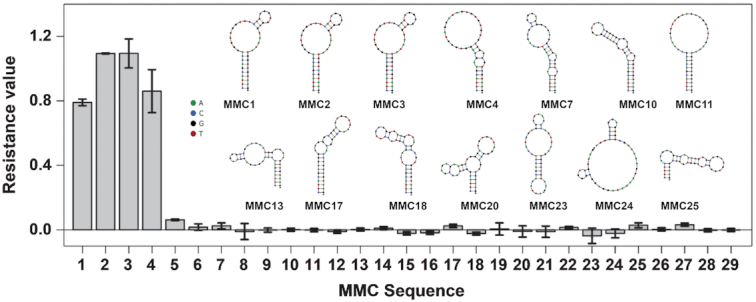
Profiling of 29 mephedrone aptamer candidates (MMC1–29) using the exonuclease profiling fluorescence assay based on the magnitude of each aptamer's resistance to exonuclease. NUPACK (45)-predicted secondary structures of binding candidates MMC1 – 4 and some non-binding sequencing are also shown. Error bars represent standard deviation of two experiments.

Next, we used the exonuclease profiling assay to determine if the 28 other aptamer candidates identified through SELEX (Supplementary Table S3, Figure S25) also bind to mephedrone. We synthesized these sequences (MMC2–29) and digested them with the exonuclease mixture in the presence of mephedrone, and recorded fluorescence time courses (Supplementary Figures S26–28). Notably, the exonuclease-based assay can differentiate binding from non-binding sequences regardless of their sequence or secondary structure. Only the four most abundant sequences in the round 11 pool (MMC1–4) showed signs of binding to mephedrone. MMC 1–4 had resistance values of 0.8–1, which indicated that these sequences can bind to mephedrone since they remained intact over the course of digestion (Figure 4). For all other sequences, the time-dependent fluorescence curves for both the target-free and target-containing samples overlapped, resulting in near-zero resistance values that indicated very weak or no mephedrone-binding capability. Continuous-injection ITC (44) results for these 28 sequences corroborated the results from the exonuclease fluorescence assay, confirming that only MMC1–4 bound to mephedrone with significant affinity (Supplementary Table S4, Figures S29–S32).

### Determining the ligand binding profile of aptamers with G-quadruplexes using the exonuclease-based assay

Finally, to assess if our assay could be used to profile the binding of ligands to aptamers with G-quadruplexes, we tested two DNA aptamers that respectively bind to synthetic cathinones (termed SCA2.1) (11) and dopamine (42). Both aptamers are believed to contain parallel or mixed G-quadruplexes (46) based on their circular dichroism (CD) spectra (42,47).

**Figure 5.**
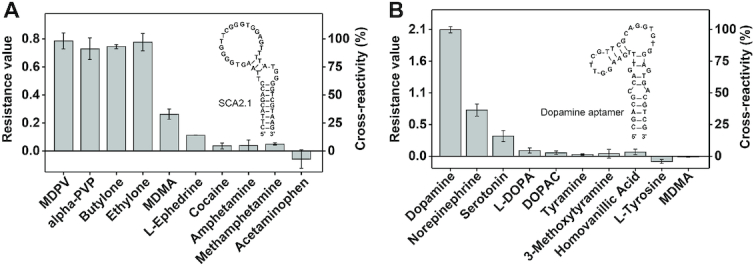
Ligand binding profile of aptamers with G-quadruplexes as determined using the exonuclease-based profiling assay. Resistance values and cross-reactivity of (**A**) SCA2.1 for each ligand relative to MDPV and (**B**) of the dopamine-binding aptamer for each ligand relative to dopamine. Error bars represent the standard deviation of three experiments.

SCA2.1 is a G-rich 46-nt DNA aptamer that binds to various illicit drugs that share the beta-keto phenethylamine core structure common to synthetic cathinones with nanomolar dissociation constants (11). We first digested SCA2.1 with T5 Exo and Exo I with and without the aptamer's primary target, MDPV, and monitored the digestion progress in a microplate format. The enzymes digested SCA2.1 within 3 h in the absence of MDPV with an exponential digestion trend. However, in the presence of MDPV, digestion of SCA2.1 was strongly inhibited (Supplementary Figure S33A), with target-concentration-dependent digestion kinetics (Supplementary Figure S33B). Our previous control experiment with MA-Mutant confirmed that enzymatic inhibition is not due to MDPV itself, but rather due to the binding of MDPV to MA-46. We therefore concluded that the inhibition of SCA2.1 digestion by the enzymes is due to the binding of MDPV to this aptamer. We then used our method to determine the binding profile of SCA2.1 to four synthetic cathinones and six structurally-similar non-target compounds (for structures see Supplementary Figure S34). In keeping with previously reported ITC data (11), SCA2.1 bound to the synthetic cathinones MDPV, alpha-PVP, butylone, and ethylone with similarly high affinity, but not to amphetamine, methamphetamine, cocaine, and acetaminophen. In addition, we observed that MDMA and l-ephedrine bound to SCA2.1, albeit with relatively weaker affinity compared to the synthetic cathinones (Figure 5A and Supplementary Figure S35). To validate these results, we performed ITC measurements of the affinity of MDMA and l-ephedrine for SCA2.1. The ITC results coincided with those from our exonuclease-based assay, showing that SCA2.1 binds MDMA and L-ephedrine with a *K*_d_ of 24.5 ± 0.9 and 64 ± 3 μM, respectively (Supplementary Figure S36).

We then tested the binding profile of a recently isolated 44-nt DNA aptamer that binds to the neurotransmitter dopamine with high affinity (42). First, we digested this aptamer in the absence and presence of dopamine with T5 Exo and Exo I. The aptamer was completely digested within 2.5 h in the absence of dopamine with an exponential digestion trend. In the presence of dopamine, digestion was greatly inhibited (Supplementary Figure S37A), and the digestion kinetics were sensitive to the concentration of dopamine (Supplementary Figure S37B). To confirm that binding of dopamine to its aptamer is solely responsible for enzyme inhibition, we digested MMC1, which has no detectable affinity for dopamine (Supplementary Figure S38A), with T5 Exo and Exo I in the presence of varying concentrations of dopamine. We observed no enzyme inhibition even with 1 mM dopamine, which indicates that the inhibition of the digestion of the dopamine-binding aptamer is specific to aptamer-target binding (Supplementary Figure S38B). We then assessed the binding profile of the dopamine-binding aptamer to 10 different ligands (for structures see Supplementary Figure S39) using our method. The results demonstrated that the aptamer bound to dopamine and, with less affinity, to norepinephrine and serotonin (Figure 5B and Supplementary Figure S40). However, the aptamer showed little or no binding to all other tested compounds including l-DOPA, 3,4-dihydroxyphenylacetic acid (DOPAC), tyramine, 3-methoxytyramine, and homovanillic acid (Figure 5B). Overall, the results of our assay matched those reported previously (29,42). We also determined that the aptamer displays no binding to other structurally similar compounds such as l-tyrosine and MDMA. Our findings with SCA2.1 and the dopamine-binding aptamer indicate that our exonuclease-based profiling assay can be used to accurately characterize the binding profiles of aptamers containing G-quadruplexes, which further extends the generality of our method to another important class of aptamer structures.

## DISCUSSION

Even as advances in DNA sequencing techniques allow for the identification of hundreds of aptamer candidates, there remains a dearth of technologies for rapid, cost-efficient, high-throughput characterization of aptamer-ligand interactions. Quantitative instrument-based methods can provide detailed binding parameters, but are ill-suited for screening large numbers of aptamer candidates. In contrast, high-throughput competition-based assays are simple to perform and do not require any specialized instrumentation, but require expensive labeling and are prone to false results. Thus, neither approach offers an optimal solution for large-scale studies of aptamer-ligand interactions or aptamer candidate screening.

In this work, we have developed a generally applicable nuclease-based approach for sensitively interrogating the binding profile of DNA aptamers that bind to small molecules. This method is based on the phenomenon that aptamer–ligand binding alters the digestion kinetics of the aptamer by the enzyme T5 Exo. We first demonstrated that the digestion of a stem-loop structured ATP aptamer by T5 Exo is inhibited a few nucleotides prior to the target-binding domain by ATP binding to the aptamer. The extent of this resistance to digestion was correlated with the strength of the aptamer–ligand interaction, and we obtained affinity and specificity results with a range of adenosine- and non-adenosine-based ligands that mirrored previous findings for this aptamer. We next demonstrated the generality of this finding with a three-way-junction structured aptamer that binds to MDPV, and showed that the addition of Exo I expedites the digestion process. We exploited this exonuclease combination in a microplate-based assay that enabled us to perform affinity analysis for up to 25 ligands simultaneously, using the DNA-staining dye SYBR Gold to monitor the digestion of the aptamer over time. By assessing the overall effect that a ligand has on the kinetics of aptamer digestion rather than relying on a single time-point, we were able to distinguish the binding affinity of the ligands with a high degree of accuracy and no ligand-related artifacts. This assay confirmed prior findings regarding the ligand-specificity of this aptamer, and revealed binding profiles for a set of compounds that were not tested before. Importantly, our assay also overcame the ligand-induced false results observed in strand-displacement assays, with findings that were confirmed with ITC. Next, we isolated a highly specific aptamer for the small-molecule drug mephedrone and evaluated its ligand-binding profile using our assay. The aptamer displayed excellent specificity for mephedrone, with the unprecedented capability to differentiate mephedrone from positional isomers 2- and 3-MMC—analogs that only differ by the position of the methyl group on the benzene ring—and methcathinone, which lacks this methyl group. Notably, this assay allowed for the rapid identification of mephedrone-binding sequences among 29 aptamer candidates in a single experiment, producing results that again matched the findings of ITC experiments. As a final demonstration of the generality of this method with respect to aptamer structure, we accurately ascertained the binding profile of two aptamers containing G-quadruplexes to a variety of small-molecule ligands.

The exonuclease-based profiling assay is robust and straightforward. In our method, T5 Exo is the enzyme that discriminates between the ligand-bound and unbound forms of the aptamer. Therefore, aptamer profiling experiments can be performed solely with T5 Exo. To speed up the assay and increase signal-to-noise ratio, Exo I can also be added to remove leftover short single-stranded DNA generated by T5 Exo. We have successfully demonstrated that same enzyme concentrations employed throughout this work (0.2 U/μl T5 Exo + 0.015 U/μl) can be used to profile the binding of aptamers with a variety of structures and ligand-binding affinities and therefore believe these enzyme concentration can serve as a good basis for future aptamer profiling experiments. In terms of the choice of buffer, pH, and ion concentrations, we recommend using the conditions that the aptamer is known to bind to its target. For example, we characterized the newly isolated aptamer MMC1 in the same buffer conditions we used for aptamer isolation. Nevertheless, our experience supports that T5 Exo and Exo I can function in different types of buffer systems (e.g. Tris and phosphate buffer) and at various ionic strengths (0–140 mM NaCl, 0.5–10 mM MgCl_2_). We are therefore confident that future users of this assay will not need to optimize any conditions and instead can use our recommended enzyme concentrations with the buffer of their choice.

Based on the findings described here as well as those in a recent work on the mechanism of DNA digestion by T5 Exo (32), we formulate a hypothetical description of the digestion process of the aptamers studied herein. To initiate digestion, T5 Exo first binds to the double-stranded region of the aptamer downstream from the 5′ blunt end. The enzyme then threads the single-stranded DNA that transiently forms due to stem breathing (48) through its helical arch, which positions the scissile phosphate over the catalytic site, resulting in cleavage of the phosphodiester bond. This is supported by our findings showing that the enzyme exonucleolytically cleaves 3–6 bases from the 5′ blunt end of the aptamers. At this point, if a ligand is not bound to the aptamer, the enzyme will continue to digest its substrate, generating mononucleotides and/or short oligonucleotide products. To rationalize the altered digestion of the aptamer when it is bound to a ligand, we presume that the enzyme has lower affinity for the ligand-bound form of the aptamer compared to the free aptamer. This could be due to steric hindrance or distortion of substrate structure imposed by ligand binding, which reduces the range of contacts that the aptamer can establish with the enzyme. Aptamer-ligand binding may also reduce the frequency of stem breathing, which prevents the enzyme from threading the aptamer. Thus, for the ligand-bound aptamer, the enzyme will continue to digest the aptamer until it is truncated to such an extent that the aptamer-ligand complex has little or no affinity for T5 Exo, causing it to disassociate from the enzyme and ceasing the digestion process.

In a related work, we previously determined that the digestion of DNA aptamers by exonuclease III (Exo III) is inhibited upon the binding of targets to aptamers (34). This finding enabled the generation of minimized structure-switching aptamers from small-molecule-binding aptamers with diverse structures. Recently, we developed an analytical method that utilizes Exo III and Exo I to achieve multiplexed small-molecule fluorescence detection (49). There, we observed that the inhibition of aptamer digestion by these exonucleases is dependent on concentration of the aptamer's target. Based on this, we were able use the quantity of the aptamer digestion product at a single point in time as a proxy for the concentration of the analyte. In this work, we described the use of T5 Exo to profile the binding of small-molecule ligands to aptamers in an accurate, rapid, high-throughput, label-free manner. This is based on our new finding that that the binding of ligands to aptamers prevents their digestion by T5 Exo, and that this inhibition correlates with the affinity of a ligand for the aptamer. Our assay entails digesting aptamers with or without ligand and monitoring the concentration of aptamer over the whole course of the digestion. We established a new metric termed ‘resistance value’, which represents the ratio of the integral of the fluorescence-time-plot curve in the presence versus the absence of ligand, to correlate aptamer ligand-binding affinity with the kinetics of enzymatic digestion without any bias related to enzyme activity, sequence, sequence motifs, or the structure of the aptamers. Our work here is the first to describe the use of exonucleases as probes to accurately profile the binding between DNA aptamers and small molecules in a label-free high-throughput manner. Although enzymes such as DNase I have been used to study the binding of proteins to DNA (50), they have limited applicability for small molecule ligands. In one of the only reports on this matter, De Rosa *et al.* used DNase I to probe the binding of aptamers to small-molecule toxin targets (51). However, they observed only subtle changes in the digestion profile of aptamers, which made it difficult to accurately determine aptamer-ligand binding strength. Disadvantageously, the assay also requires fluorophore labeling of the aptamer and electrophoretic separation, which makes it largely unsuitable for high-throughput screening of binding interactions. In contrast, our T5 Exo-based assay is highly sensitive to the binding of small molecules to aptamers and can be generally applied to aptamers of varying sequence and structure as well as ligands of differing physicochemical properties. In addition, our assay does not require aptamer engineering or foreknowledge of aptamer target-binding domains.

In conclusion, our assay offers the novel capability to assess the binding of hundreds of DNA aptamer-small-molecule ligand pairs simultaneously with high accuracy, which should greatly accelerate the identification of the most suitable aptamers for use in real-world applications. In the context of aptamer–ligand profiling, we believe our method has highly advantageous features compared to existing methods similar to the benefits that high-throughput sequencing offers relative to the traditionally used Sanger sequencing method. For SELEX, although both techniques provide the same information (aptamer sequence), the latter can provide a higher volume of data at a lower cost and less time, which can be used, for example, to ascertain more comprehensive information on aptamer families, structural motifs, and binding profiles. Similarly, our assay can rapidly identify the binding spectra of aptamers to a wide range of compounds to select aptamers with optimal binding affinities and specificities from a large number of candidates for use in real applications. In future applications, if any ligand is found to affect the fluorescence of SYBR Gold, other fluorescent nucleic-acid-binding dyes with varying excitation/emission wavelengths can be used, such as SYBR Green I or Quantifluor (52). Additionally, our assay may show potential for assessing the binding profiles of aptamers with other chemistries, but this would warrant a future systematic study of digesting modified aptamer constructs with altered bases or sugars at both 5′ and 3′ termini as well as interior nucleobases with T5 Exo or other nucleases. Nevertheless, given the generality of our assay for DNA aptamers with different secondary structures such as stem–loops, three-way-junctions, and G-quadruplexes, as well as its compatibility with a wide variety of ligands with vastly different physicochemical properties, we believe this method could readily be automated with a liquid-handling system to even further expedite the aptamer characterization process.

## Supplementary Material

gkaa849_Supplemental_File
