# Supplementary material for: Label-free profiling of DNA aptamer-small molecule binding using T5 exonuclease
Source: Nucleic Acids Res. 2020 Oct 14;48(20):e120. doi: 10.1093/nar/gkaa849 (PMC7672472; doi:10.1093/nar/gkaa849)
Supplement: gkaa849_Supplemental_File [file gkaa849_supplemental_file.pdf]

SUPPLEMENTARY INFORMATION

Label-Free Profiling of DNA Aptamer-Small Molecule Binding Using T5 Exonuclease

Obtin Alkhamis†, Weijuan Yang†, Rifat Farhana, Haixiang Yu and Yi Xiao*

Department of Chemistry and Biochemistry, Florida International University, 11200 SW 8th Street, Miami, FL, USA, 33199

†These authors contributed equally to this work.
*Corresponding author: yxiao2@fiu.edu

MATERIALS AND METHODS

Reagents and materials. T5 Exonuclease (T5 Exo; 10 U/µl) and Exonuclease I (Exo I; 20 U/µl) were purchased from New England Biolabs. Bovine serum albumin was purchased from Sigma Aldrich. Adenosine 5'-triphosphate (ATP) disodium salt hydrate was purchased from MP Biomedicals. Adenosine 5'-diphosphate (ADP) sodium salt, adenosine 5'-monophosphate (AMP) disodium salt, and adenosine were purchased from Sigma Aldrich. Uridine 5'-triphosphate (UTP), cytidine 5'-triphosphate (CTP), and guanidine 5'-triphosphate (GTP) were purchased from Thermo Fisher Scientific as 100 mM solutions buffered to ~pH 7.4 with Tris base. 3,4-methylenedioxypyrovalerone (MDPV), naphyrone, methylone, 2-methylmethcathinone, 3-methylmethcathinone, 4-methylmethcathinone, methedrone, 3-fluoromethcathinone, 4-fluoromethcathinone, ethylone, α-pyrrolidinopentiophenone, cathinone, methcathinone, butylone, ethcathinone, N,N-dimethylmethcathinone, 4'-methyl-α-pyrrolidinobutiophenone, pyrovalerone, 3',4'-methylenedioxy-α-pyrrolidinopropiophenone, and pentylone were purchased from Cayman Chemical as hydrochloride (HCl) racemic salts. 3',4'-methylenedioxymethamphetamine HCl, acetyl fentanyl HCl, and morphine sulfate hydrate were purchased from Cayman Chemical. Acetaminophen, benzocaine, chlorpromazine HCl, cocaine HCl, diphenhydramine HCl, dopamine HCl, (-)-ephedrine HCl, lidocaine HCl, amphetamine HCl, methamphetamine HCl, procaine HCl, pseudoephedrine HCl, serotonin HCl, quinine hemisulfate monohydrate, (-)-norepinephrine bitartrate, L-DOPA, 3,4-dihydroxyphenylacetic acid (DOPAC), tyramine HCl, 3-methoxytyramine, homovanillic acid, and L-tyrosine were purchased from Sigma Aldrich. Formamide, SYBR Gold, streptavidin-coated agarose resin (capacity: 1-3 mg biotinylated BSA/ml resin), One Shot Chemically Competent *E. coli*, TOPO TA cloning kit, and PureLink Quick Plasmid Miniprep Kit were purchased from Thermo Fisher Scientific. 500 µL micro-gravity columns were purchased from Bio-Rad. GoTaq Hot Start Colorless Master Mix was purchased from Promega. 3 kDa cut-off spin filters were purchased from Millipore. All other chemicals were purchased from Sigma-Aldrich unless otherwise specified. All oligonucleotides used in this work are listed in **Table S1**.

SELEX procedure. The isolation of aptamers was carried out following a previously reported library-immobilized SELEX protocol.[2] Selection conditions are listed in **Table S2**. The initial single-stranded DNA library consisted of ~6 × 10^{14} oligonucleotides. Each library strand is stem–loop structured and 73 nt in length, with a randomized 30-nt loop flanked by a pair of 8-nt stem-forming sequences and two primer-binding regions. For each round of SELEX, the library/pool was mixed with biotinylated capture strands at a molar ratio of 1:5 in selection buffer (10 mM Tris–HCl, pH 7.4, 20 mM NaCl, 0.5 mM $MgCl_2$), heated at

95 °C for 10 min and cooled at room temperature for over 30 min to ensure hybridization between library and capture strands. A micro-gravity column (0.5 ml) was prepared by adding 250 µl of streptavidin-coated agarose beads followed by three washes with 250 µl of selection buffer. About 250 µl of cDNA-library solution was then flowed through the micro-gravity column three times in order to load the library onto the agarose beads, and the column was then washed 10 times with selection buffer. Then, 250 µl of target (mephedrone) dissolved in selection buffer was added to the column. Strands that bound to the target were displaced from the biotinylated cDNA and released into solution, and the eluent containing these strands was collected. This process was performed three times in total, and all eluents were combined (750 µl total). The resulting pool was concentrated via centrifugation using a 3 kDa cut-off spin filter. 100 µl of this concentrated pool was then mixed with 1 ml of GoTaq Hot Start Colorless Master Mix with 1 µM forward primer (**Supplementary Table S1**, FP) and 1 µM biotinylated reverse primer (**Supplementary Table S1**, RP-bio) to PCR amplify the pool. Amplification was performed in a BioRad C1000 thermal cycler with conditions as follows: 2 min at 95 °C; 13 cycles of 95 °C for 15 s, 58 °C for 30 s, and 72 °C for 45 s, followed by 5 min at 72 °C. The optimal number of amplification cycles was determined empirically. Amplification of the enriched pool and the absence of byproducts were confirmed using 3% agarose gel electrophoresis. If byproducts with differing lengths from the original library strands were observed, the pool was purified with a 4% agarose gel and the 73-nt products were recovered by silica column as reported previously.[2] Prior to the 6th round of selection, error-prone PCR was performed to increase pool diversity and reduce the prevalence of cross-reactive sequences (see "Error-prone PCR protocol" below). To generate single-stranded DNA from the PCR products, a fresh micro-gravity column was prepared containing 250 µl streptavidin-coated agarose beads, as described above. The amplified pool was then flowed through the column three times to conjugate the pool to the beads. Afterward, the column was washed six times with 250 µl of separation buffer (10 mM Tris–HCl, 20 mM NaCl, pH 7.4). The column was then capped, and 300 µl of a 0.2 M NaOH solution was added to the column and incubated for 10 min to generate single-stranded DNA, after which the eluent was collected. An additional 100 µl of 0.2 M NaOH was added to elute residual library strands from the column. Both eluents were combined and neutralized with 0.2 M HCl, and the pool was then concentrated via centrifugation with a 3 kDa cut-off spin filter.

Counter-SELEX procedure. For every round after the first, counter-SELEX was performed before positive selection to remove cross-reactive sequences. The library-immobilized column was washed with 250 µl of counter-target(s) in selection buffer to remove non-specific DNA strands. This process was performed four times for Rounds 2–5 and three times for Rounds 6-11. Afterward, the column was washed 60 times with 250 µl selection buffer to wash away non-specific binders in preparation for positive selection. From round 6 to round 11, pre-incubation counter-SELEX was performed before immobilizing the cDNA-library complex onto beads. Methedrone and methylone dissolved in 250 µl of selection buffer was added to the cDNA-library complex after the hybridization step, incubated on an end-over-end rotator for 60 min, and stored at 4 °C overnight.

Error-prone PCR. Prior to the 6th round of SELEX, error-prone PCR was performed in a mutagenic mixture (final concentrations: 10 mM Tris-HCl (pH 8.3), 50 mM KCl, 7 mM $MgCl_2$, 0.5 mM $MnCl_2$, 0.2 mM dGTP, 0.2 mM dATP, 1 mM dCTP, 1 mM dTTP, 1 µM RP-bio, 1 µM FP, 0.05 U/µL Taq DNA polymerase) with 2 ng/µl of the 5th round SELEX pool as the template. First, 16 tubes of reagent were prepared in which each tube contained 10 µl of 10× PCR buffer (100 mM Tris–HCl, 500 mM KCl, 70 mM $MgCl_2$, pH 8.3), 0.2 µl of 100 mM dGTP, 0.2 µl of 100 mM dATP, 1.0 µl of 100 mM dCTP, 1.0 µl of 100

mM dTTP, 1.0 µl of 100 µM RP-bio, 1.0 µl of 100 µM FP and 72.6 µl of water, and these were stored on ice. 10 µl of 20 ng/µl DNA template collected from the 5th selection pool was added to the first tube, which was placed in a Bio-Rad C1000 thermal cycler for the first cycle of the following program: 1 min at 95 °C; 2 min at 58 °C; 3 min at 72°C. Once the PCR program reached the annealing temperature (58 °C) in the first cycle, 2 µl of 25 mM freshly prepared $MnCl_2$ solution and 1 µl of 5 U/µl Taq DNA polymerase were added to the PCR reaction tube and mixed by pipette. Another 3 cycles of amplification were then performed as follows: 95°C for 1 min, 58°C for 1 min, 72°C for 3 min. The first tube was then stored on ice after removing 10 µl of the PCR product and transferring it to the second tube for amplification with the same error-prone PCR program as the first tube. The process was repeated for a total of 16 serial transfer steps. These 16 tubes of PCR product were analyzed using 3% agarose gel electrophoresis and combined for separation. The whole error-prone PCR pool was then used as the input DNA library pool for the 7th round of SELEX.

Cloning and sequencing. After 11 rounds of SELEX, the final enriched pool was PCR amplified with unlabeled forward and reverse primers (**Supplementary Table S1**, FP and RP) under the conditions described above, with a prolonged 30-min extension step at 72 °C to add a poly-A tail. The PCR product was cloned into *E. coli* using the TOPO TA cloning kit (Invitrogen). Fifty colonies were randomly picked and sequenced at the Florida International University DNA Core Facility. Sequence alignments were carried out using BioEdit and the aligned sequences were listed in **Table S3**.

Isothermal titration calorimetry (ITC). All experiments were performed at 23 ºC in the aptamers' respective reaction buffers with a MicroCal iTC200 instrument (Malvern). A 40 µl quantity of ligand was loaded into the syringe and titrated into a cell containing 20 µM aptamer. The concentration of ligand used in each experiment is listed in **Table S4**. Twenty 2 µl injections were performed, with an initial purge injection of 0.4 µl. Each injection was spaced out by 150 sec with an initial 60-sec delay before the first titration. The heat released upon each addition of ligand was recorded over time. The raw data were first corrected based on the heat of dilution of target, and then analyzed with the MicroCal analysis kit integrated into Origin 7 software with a binding model. For ITC experiments involving MA with ethylone and MMC1 with methedrone, methcathinone, ethylone, and methylone, two sets of titrations were performed back-to-back, and the data obtained from both titrations were combined together. For fitting of ATP-33 titrations, a sequential binding model was used with two binding sites. For fitting of MA and SCA2.1 titrations, a one-site binding model was used. For fitting of MMC1 titrations, a one-site binding model was used, but the concentration of ligand used for calculation was half of the actual concentration of ligand employed. This is based on the assumption that the aptamer binds to only one enantiomer of synthetic cathinones (which were all used as a racemic mixtures) and has negligible affinity for the other. With this treatment, the binding stoichiometry for mephedrone-MMC1 binding is close to 1, which is the most common binding stoichiometry for aptamer-ligand binding. For screening of MMC aptamers binding to mephedrone, the continuous-injection method was performed. This method serves as a rapid approach for screening receptor-ligand interactions.[1] For these experiments, desalt purity DNA was used. After a pre-injection delay of 60-seconds, 40 µl of 1000 µM mephedrone was injected into a cell containing 20 µM aptamer over a span of 15 min followed by a 5-min post-injection wait period. The data was analyzed using the MicroCal analysis kit. The heat released by ligand titration to buffer were subtracted from all data sets. Pre- and post-injection regions were omitted from the final binding curve.

References:

(1) Markova, N.; Hallén, D. The Development of a Continuous Isothermal Titration Calorimetric Method for Equilibrium Studies. *Anal. Biochem.* **2004**, *331*, 77–88.

(2) Yang, W.; Yu, H.; Alkhamis, O.; Liu, Y.; Canoura, J.; Fu, F.; Xiao, Y. In Vitro Isolation of Class-Specific Oligonucleotide-Based Small-Molecule Receptors. *Nucleic Acids Res.* **2019**, 47(12), e71.

Table S1. List of oligonucleotides used in this work.

Sequence ID	Sequence (5' – 3')
ATP-33	CGCACCTGGGGGAGTATTGCGGAGGAAGGTGCG
ATP-33-M	CGCACCTGGGGAAGTATTGCGGTGGAAGGTGCG
ATP-28	CTGGGGGAGTATTGCGGAGGAAGGTGCG
MA	CTTACGACTCAGGCATTTTGCCGGGTAACGAAGTTACTGTCGTAAG
MA-Mutant	CTTACGACGCAGGCATTTTGCCGGGTAACGAAGTTACTGTCGTAAG
MA-41	GACTCAGGCATTTTGCCGGGTAACGAAGTTACTGTCGTAAG
MA-FAM	/56-FAM/TGGCAGAACTTACGACTCAGGCATTTTGCCGGGTAACGAAG TTACTGTCGTAAG
Dab-15	GTCGTAAGTTCTGCC/3Dab/
MMC1	CTTACGACCAGGGTTGGTTTCATCGGTGGTGTAATATGGTCGTAAG
MMC1-Mutant	CTTACGACCAGGGTTGGTTTCACCGGTGGTGTAATATGGTCGTAAG
MMC1-42	CGACCAGGGTTGGTTTCATCGGTGGTGTAATATGGTCGTAAG
MMC2	CTTACGACCAGGGTTGGTCTCATCGGTGGTGTAATATGGTCGTAAG
MMC3	CTTACGGCCAGGGTTGGTCTCATCGGTGGTGTAATATGGTCGTAAG
MMC4	CTTACGAAGCAGGTGGGTAGGATAGTGTGTGTGCTGTCGTCGTAAG
SCA2.1	CTTACGACCTTAAGTGGGGTTCGGGTGGAGTTTATGGGGTCGTAAG
Dopamine Aptamer	CGACGCCAGTTTGAAGGTTCGTTCGCAGGTGTGGAGTGACGTCG
DNA Library	CGAGCATAGGCAGAACTTACGAC(N_{30})GTCGTAAGAGCGAGTCATTC
cDNA-bio	TTTTTGTCGTAAGTTCTGCCATTTT/Bio/
Forward primer (FP)	CGAGCATAGGCAGAACTTAC
Reverse primer-bio (RP-bio)	/Bio/GAATGACTCGCTCTTACGAC
Reverse primer (RP)	GAATGACTCGCTCTTACGAC

/Bio/ = Biotin attached via 3-carbon linker to the terminal nucleotide
/56-FAM/ = 5' fluorescein modification
/3Dab/ = 3' Dabcyl modification

Table S2. Detailed SELEX conditions for the isolation of mephedrone-binding aptamers.

Round #	Pool size (pmole)	Pre-incubation	Counter Targets								Target (μM)
			Group 1	Group 2	Group 3	Group 4	Group 5	Group 6	Group 7	Group 8	
1	1000	N/A									1000
2	290	N/A	COC, PSE, ACM, PRC, CAF, SUC (200 μM each) x 1.00 mL	MPBP, PYR, MPHP (200 μM each) x 1.00 mL	α-PVP, DMC, ETY, BTY (200 μM each) x 1.00 mL	2-MMC, 3-FMC, 3-MMC, MCT, CAT (200 μM each) x 1.00 mL	4-FMC, MTD, MTL (200 μM each) x 1.00 mL	N/A	N/A	NA	500
3	334		COC, PSE, ACM, PRC, CAF, SUC (250 μM each) x 1.00 mL	MPBP, PYR, MPHP (250 μM each) x 1.00 mL	α-PVP, DMC, ETY, BTY (250 μM each) x 1.00 mL	2-MMC, 3-FMC, 3-MMC, MCT, CAT (250 μM each) x 1.00 mL	4-FMC, MTD, MTL (250 μM each) x 1.00 mL				250
4	250		COC, PSE, ACM, PRC, CAF, SUC (500 μM each) x 1.00 mL	MPBP, PYR, MPHP (500 μM each) x 1.00 mL	α-PVP, DMC, ETY, BTY (500 μM each) x 1.00 mL	2-MMC, 3-FMC, 3-MMC, MCT, CAT (500 μM each) x 1.00 mL	4-FMC, MTD, MTL (500 μM each) x 1.00 mL				200
5	200		COC, PSE, ACM, PRC, CAF, SUC (500 μM each) x 1.00 mL	MPBP, PYR, MPHP (500 μM each) x 1.00 mL	α-PVP, DMC, ETY, BTY (500 μM each) x 1.00 mL	2-MMC, 3-FMC, 3-MMC, MCT, CAT (500 μM each) x 1.00 mL	4-FMC, MTD, MTL (500 μM each) x 1.00 mL				200
Error-prone PCR											
6	535	MTL,MTD (2000 μM each)	PSE, ACM, PRC (500 μM each) x 0.75 mL	α-PVP, DMC, BTY (500 μM each) x 0.75 mL	3-FMC, MCT (500 μM each) x 0.75 mL	CAT (1000 μM) x 0.75 mL	ETY (1000 μM) x 0.75 mL	4-FMC(1000 μM) x 0.75 mL	MTL (2000 μM) x 0.75 mL	MTD (2000 μM) x 0.75 mL	100
7	200		COC, CAF, SUC (500 μM each) x 0.75 mL	MPBP, PYR, MPHP (500 μM each) x 0.75 mL	2-MMC, 3-MMC (500 μM each) x 0.75 mL						50
8	100		PSE, ACM, PRC (500 μM each) x 0.75 mL	α-PVP, DMC, BTY (500 μM each) x 0.75 mL	3-FMC, MCT (500 μM each) x 0.75 mL						50
9	100		COC, CAF, SUC (500 μM each) x 0.75 mL	MPBP, PYR, MPHP (500 μM each) x 0.75 mL	2-MMC, 3-MMC (500 μM each) x 0.75 mL						50
10	538	MTL,MTD (2000 μM each)	PSE, ACM, PRC (500 μM each) x 0.75 mL	α-PVP, DMC, BTY (500 μM each) x 0.75 mL	3-FMC, MCT (500 μM each) x 0.75 mL	CAT (1000 μM) x 0.75 mL	ETY (1000 μM) x 0.75 mL	4-FMC(1000 μM) x 0.75 mL	MTL (2000 μM) x 0.75 mL	MTD (2000 μM) x 0.75 mL	100
11	200		COC, CAF, SUC (500 μM each) x 0.75 mL	MPBP, PYR, MPHP (500 μM each) x 0.75 mL	2-MMC, 3-MMC (500 μM each) x 0.75 mL						50

Cocaine (COC), pseudoephedrine (PSE), acetaminophen (ACM), procaine (PRC), caffeine (CAF), sucrose (SUC), pyrovalerone (PYR), ethylone (ETY), butylone (BTY), methcathinone (MCT), cathinone (CAT), methedrone (MTD), methylone (MTL), Dimethylcathinone (DMC), α-Pyrrolidinopentiophenone (α-PVP), 4'-Methyl-α-pyrrolidinobutiophenone (MPBP), 4'-Methyl-α-pyrrolidinohexiophenone (MPHP), 3-fluoromethcathinone (3-FMC), 4-fluoromethcathinone (4-FMC), 2-methylmethcathinone (2-MMC), 3-methylmethcathinone (3-MMC)

Table S3. Sequences and prevalence of clones from the Round 11 pool determined via Sanger sequencing.

Sequence	Sequence (5' to 3')	Count
1	CTTACGACCAGGGTTGGTTTCATCGGTGGTGTAATATGGTCGTAAG	15
2	CTTACGACCAGGGTTGGTCTCATCGGTGGTGTAATATGGTCGTAAG	3
3	CTTACGGCCAGGGTTGGTCTCATCGGTGGTGTAATATGGTCGTAAG	2
4	CTTACGAAGCAGGTGGGTAGGATAGTGTGTGTGCTGTCGTCGTAAG	3
5	CTTACCGACTATATGGGCAGTATCGTCTAGTGCCTAAGGGTCGTAAG	2
6	CTTACAACCCAATACAAAAGCCAGCAGATATAGTGCGGGTCGTAAG	1
7	CTTACAACCCAGTGCAGCGCCGGGAGGGAGAGGCTGAGGTCGTAAG	1
8	CTTACCACAGACCAAAAGCCTGAATTAGAGTGTTGCGTGTCGTAAG	1
9	CTTACCACTCGACCCAGAGAGGTGAAACAGGTGTCTTCGTCGTAAG	1
10	CTTACGACAAGTGGCATCGAGTTACCGGTGCGCGCTGTGTCGTAAG	1
11	CTTACGACACGCCAAAGTTTGTAGACGGTTAGAAACGTGTCGTAAG	1
12	CTTACGACCAAGCAGCGTGATAGGGTCACGTGGGGATAGTCGTAAG	1
13	CTTACGACCCAAGCGAGGCAGGGCCGACCACTGGAGTAGTCGTAAG	1
14	CTTACGACCGCATCAGCGAGGAACCGTGTTAATCGACGGTCGTAAG	1
15	CTTACGACCTGGTGAGGGATAAGGATGAGACCTGCCGCGTCGTAAG	1
16	CTTACGACGATAGGACAACTATTGGTGGTCCGCACTGCGTCGTAAG	1
17	CTTACGACGGGATGGGCTGACTGTTTAATCAGGCACCTGTCGTAAG	1
18	CTTACGACTAAGCCGTCCCTTCCTGGTACGTTAGCCGTGTCGTAAG	1
19	CTTACGGCAGTGACATTAGGTAGACTCTATGGCACATAGTCGTAAG	1
20	CTTACGGCTACCGAGAGAAGCTGGGCTGTGTTAGGGCCGTCGTAAG	1
21	CTTACACAGGCAGCCGGGAGGAACCTGACCAAGAGGCGTCGTAAG	1
22	CTTACACCCTGGTGGCTAGTAGAGTTATTGGAAATGGGTCGTAAG	1
23	CTTACACGACCATCATCTAGGCTAACCCATTTCAGTGGTCGTAAG	1
24	CTTACACGCAGTGTCGAAAAAGTAAAATAGGAGGCGGGTCGTAAG	1
25	CTTACACGCGGACAACTGTGCTGATGTGGGCTCGGTGGTCGTAAG	1
26	CTTACGACCCGCAGAGGGCTACTAGGCTAGGTTGTTGGTCGTAAG	1
27	CTTACGACGTGGCGAGCATAATAAAGCACGTCCTAAAGTCGTAAG	1
28	CTTACCGGTCATTGTTGCCGCCTAAGTAGTGGTGGCGTCGTAAG	1
29	CTTACCAGCGAGTAGTTACCTTCTGAGAGGCTGTCGTAAG	1

Table S4. Aptamer and ligand concentration used for ITC experiments.

Aptamer	Aptamer concentration (µM)	Ligand	Ligand concentration (µM)
ATP-28	20	ATP	800
MA	20	MDPV	1000
MA	20	Ethylone	1000
MA	20	MDMA	1000
MA	20	Serotonin	1000
MA	20	Lidocaine	1000
MA	20	Methamphetamine	1000
MA	20	Acetyl fentanyl	1000
MA	20	Morphine	1000
MA	20	Chlorpromazine	750
MA	20	Diphenhydramine	1000
MA-41	20	MDPV	2000
MA-Mutant	20	MDPV	1000
MMC1	20	Mephedrone	2000
MMC1	20	Methedrone	3000
MMC1	20	Methcathinone	4000
MMC1	20	Ethylone	4000
MMC1	20	Methylone	4000
MMC1	20	2-MMC	4000
MMC1	20	3-MMC	4000
MMC1	20	4-FMC	4000
MMC1	20	Dopamine	1000
MMC1-42	20	Mephedrone	2000
MMC1-Mutant	20	Mephedrone	2000
Continuous injection ITC: MMC1-29	20	Mephedrone	1000
SCA2.1	20	MDMA	700
SCA2.1	20	L-ephedrine	1000

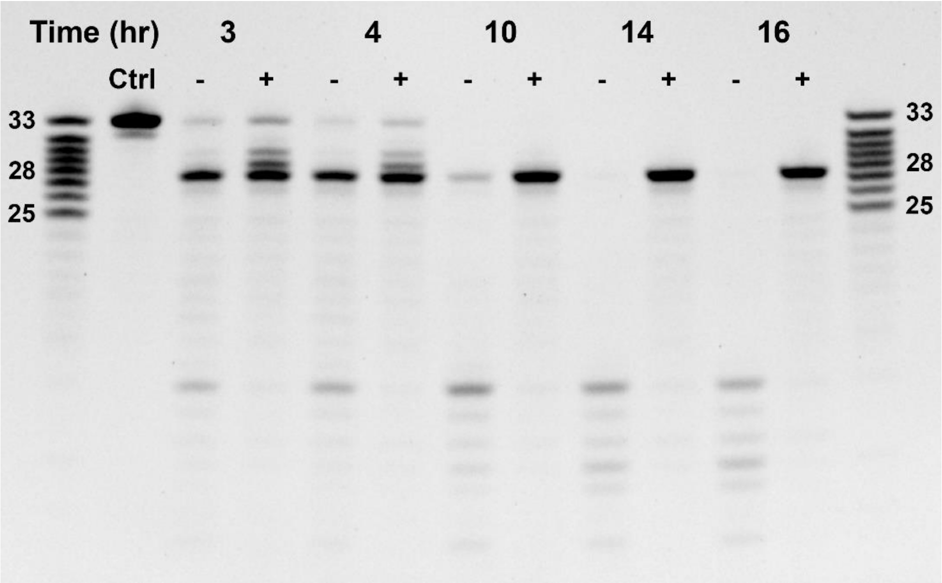

Figure S1. Time course digestion of ATP-33 by 0.2 U/μl T5 Exo T5 in the absence (-) and presence (+) of 250 μM ATP analyzed by PAGE. Buffer: 10 mM Tris-HCl (pH 7.4), 10 mM $MgCl_2$.

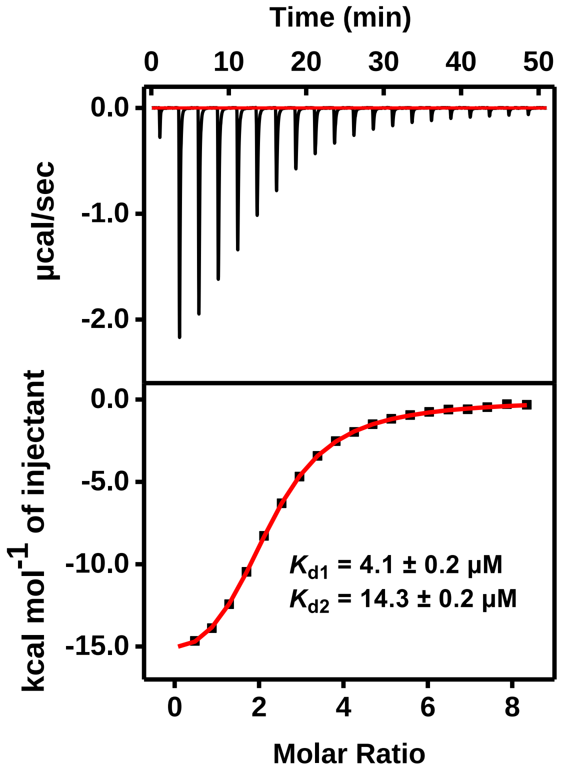

Figure S2. Characterization of the target-binding affinity of ATP-28 to ATP using ITC. The top panel presents raw data showing the heat generated from each titration of ATP to ATP-28, while bottom panel show the integrated heat of each titration after correcting for dilution heat of the titrant. ITC data were fitted with a sequential binding model with two binding sites.

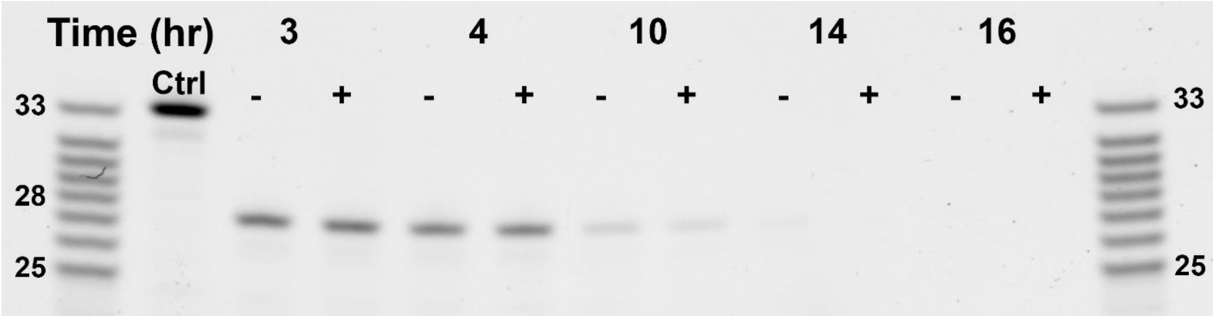

Figure S3. Time course digestion of ATP-Mutant by T5 Exo in the absence (-) or presence (+) of 250 μM ATP analyzed by polyacrylamide gel electrophoresis (PAGE).

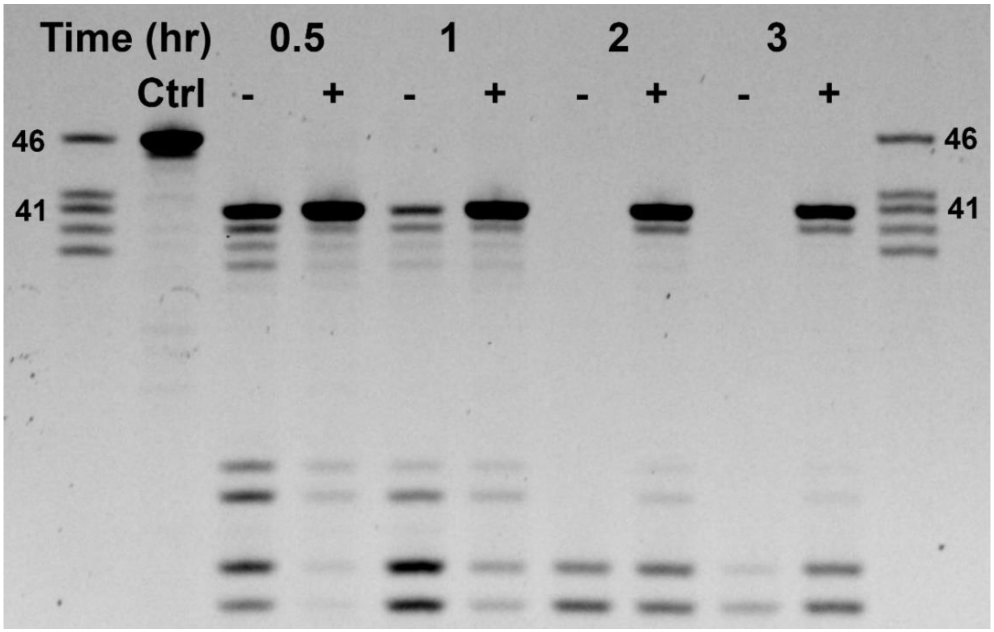

Figure S4. Time course digestion of MA by T5 Exo in the absence (-) and presence (+) of 250 μM MDPV analyzed by PAGE.

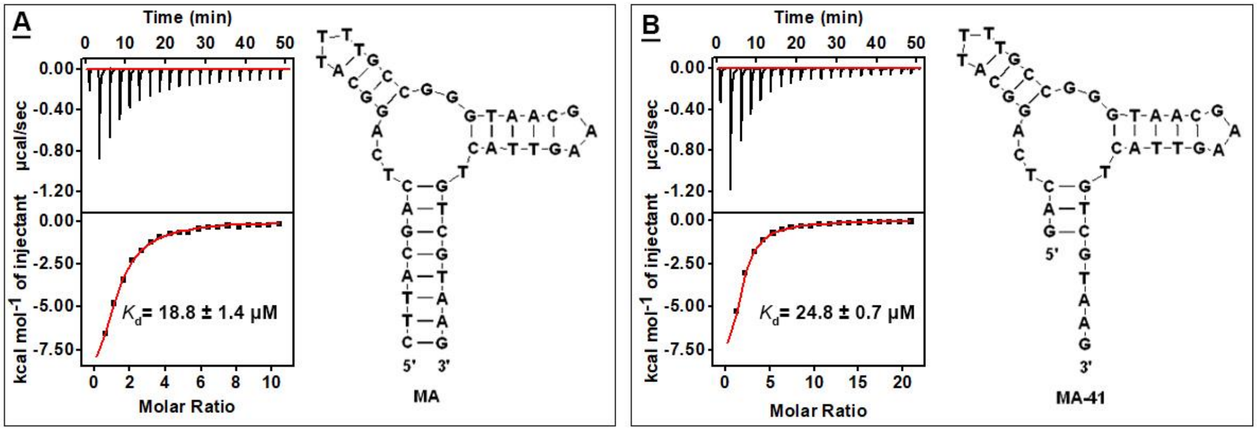

Figure S5. Characterization of the target-binding affinity of (**A**) MA and (**B**) MA-41 using ITC. Top panels present raw data showing the heat generated from each titration of MDPV to either aptamer, while bottom panels show the integrated heat of each titration after correcting for dilution heat of the titrant. ITC data were fitted with a one-site binding model. NUPACK-predicted secondary structures of the aptamers is provided.

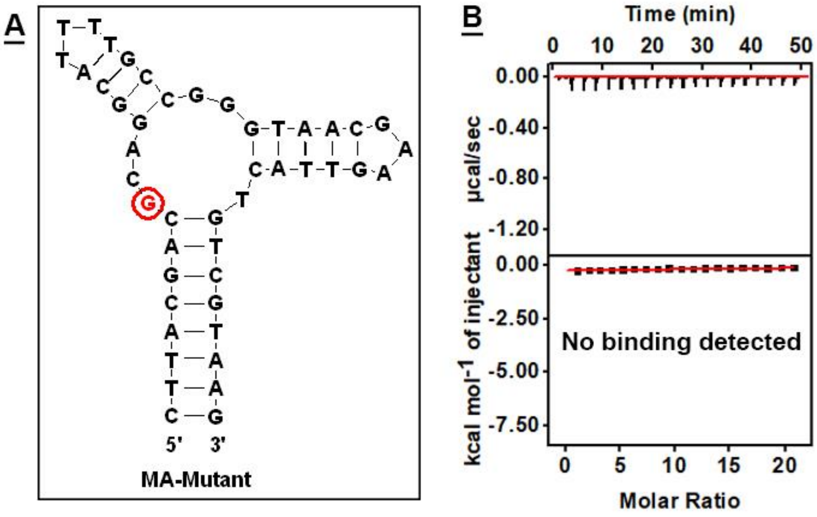

Figure S6. Characterization of the MDPV-binding affinity of MA-Mutant using ITC. (**A**) The NUPACK-predicted structure of MA-Mutant, which was engineered by mutating the T at nucleotide 9 of the parent aptamer (MA) to G (highlighted in red). (**B**) Characterization of the target-binding affinity of MA-Mutant using ITC. Top panels present raw data showing the heat generated from each titration of MDPV to the aptamer, while the bottom panels show the integrated heat of each titration after correcting for dilution heat of the titrant. ITC data were fitted with a one-site binding model.

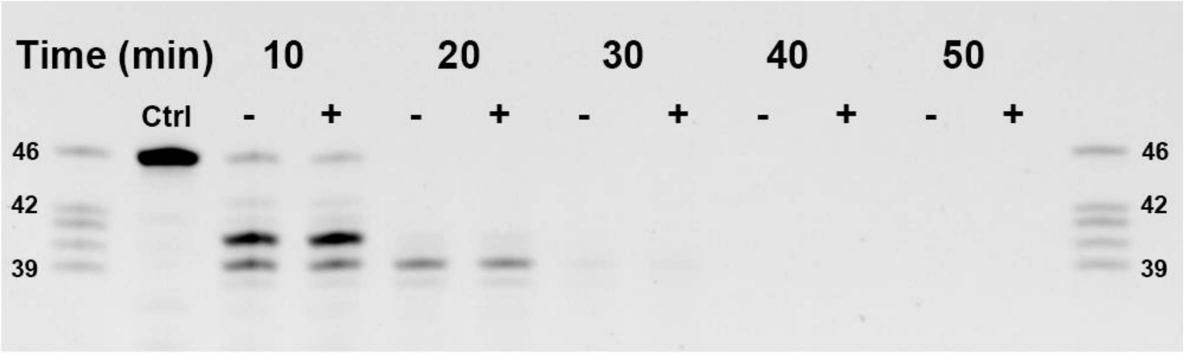

Figure S7. Time course digestion of MA-Mutant by T5 Exo and Exo I in the absence (-) and presence (+) of 250 µM MDPV analyzed using PAGE.

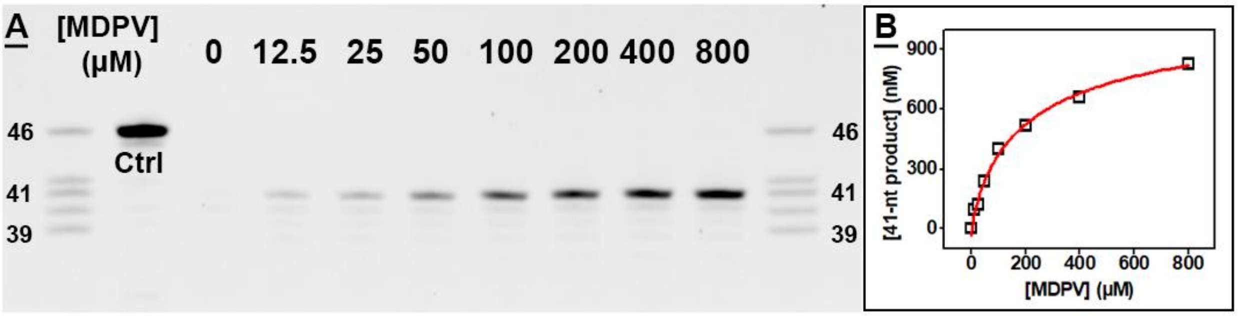

Figure S8. Calibration curve for exonuclease digestion of MA. (**A**) PAGE analysis of the digestion of MA by T5 Exo and Exo I after 40 min in the absence or presence of different concentrations of MDPV. (**B**) Amount of 41-nt product produced as a function of the concentration of MDPV. DNA concentrations were calculated relative to their respective ladder band.

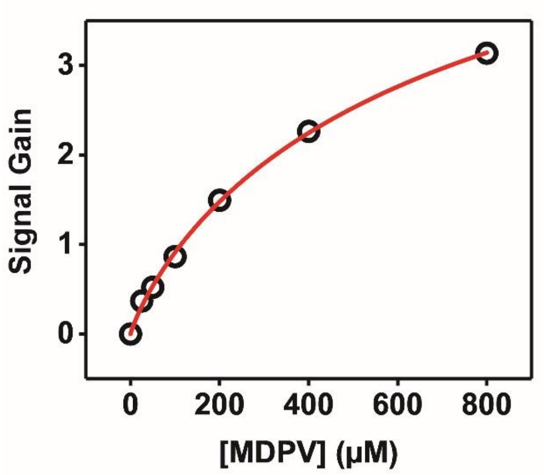

Figure S9. Fluorescence calibration curve for the exonuclease-based profiling assay with MA in the presence of 0, 25, 50, 100, 200, 400, 800 μM MDPV. Signal gain was calculated at 40 min using the equation $(F - F_0)/F_0$, where F and F_0 represent fluorescence intensity in the presence and absence of target, respectively.

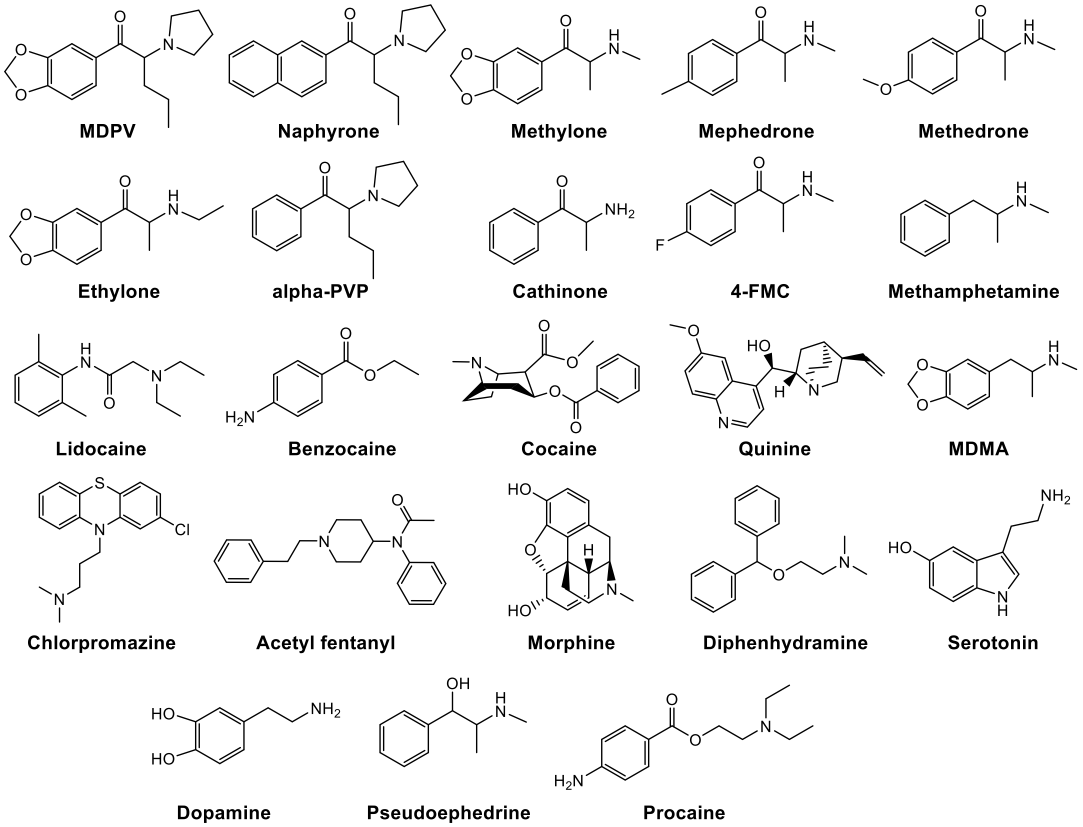

Figure S10. The chemical structures of ligands tested in the exonuclease-based profiling assay with MA-46.

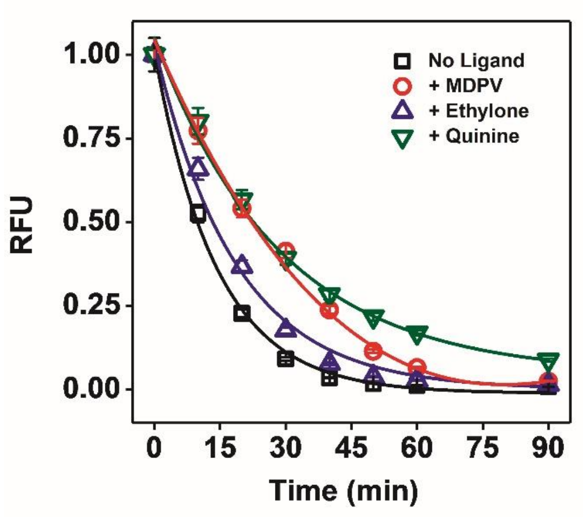

Figure S11. Time course of MA digestion by T5 Exo and Exo I. Relative fluorescence at various time points in the absence or presence of 400 μM MDPV, ethylone, or quinine. Error bars represent the standard deviation of three experiments.

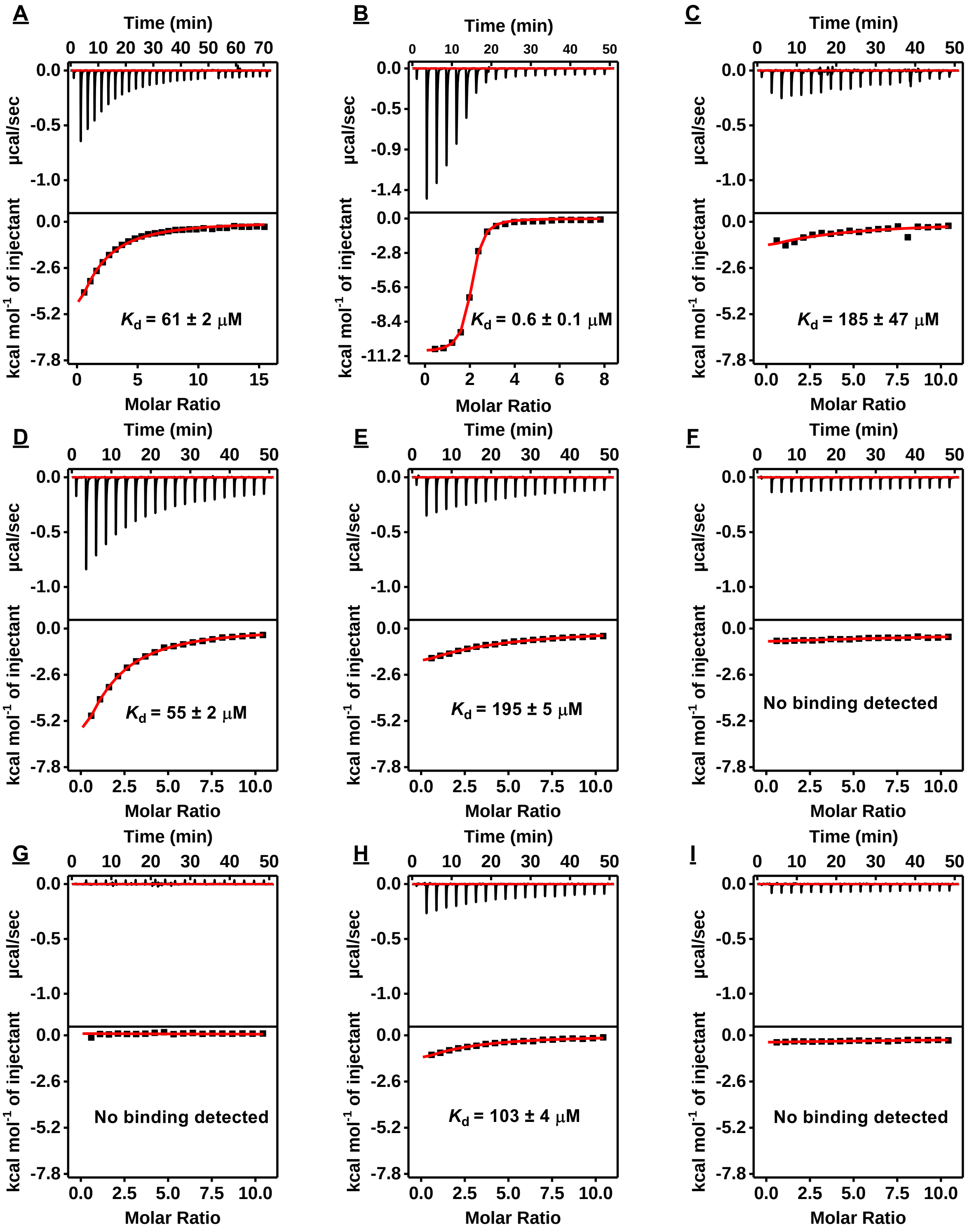

Figure S12. Characterization of the affinity of MA to a variety of ligands using ITC. Top panels present raw data showing the heat generated from each titration of (**A**) ethylone, (**B**) chlorpromazine, (**C**) acetyl fentanyl, (**D**) serotonin, (**E**) diphenhydramine, (**F**) lidocaine, (**G**) morphine, (**H**) MDMA, and (**I**) methamphetamine to MA, while bottom panels show the integrated heat of each titration after correcting for dilution heat of the titrant. ITC data were fitted with a one-site binding model.

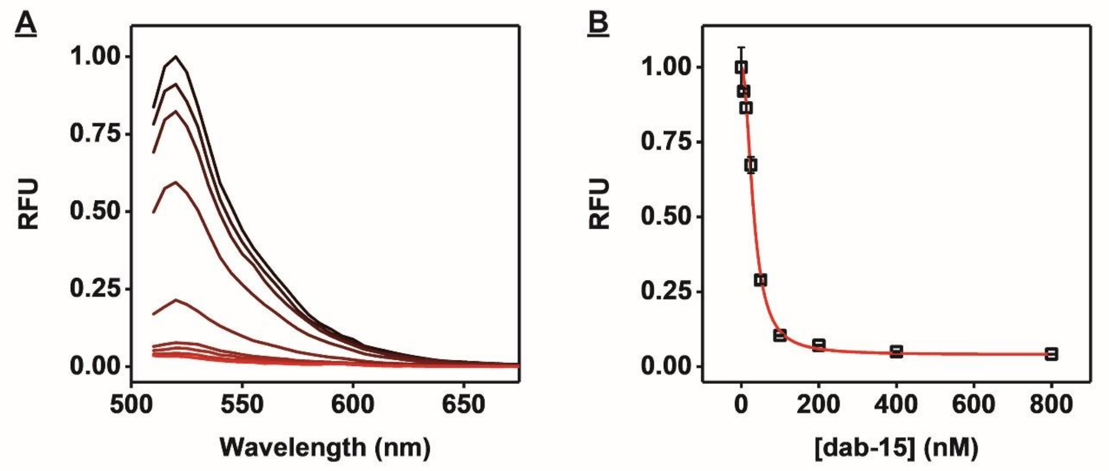

Figure S13. Optimizing dabcyl-labeled complementary DNA (dab-15) concentration for the strand-displacement assay with fluorescein-labeled MA (MA-FAM). (**A**) Spectra of 50 nM MA-FAM with increasing concentrations of dab-15 (black to red color gradient: 0, 6.3, 12.5, 25, 50, 100, 200, 400, 800 nM). (**B**) Fluorescence of MA-FAM at emission wavelength (520 nm) as a function of dab-15 concentration. [dab-15] > 200 nM results in >90% quenching of fluorescence. RFU = relative fluorescence units.

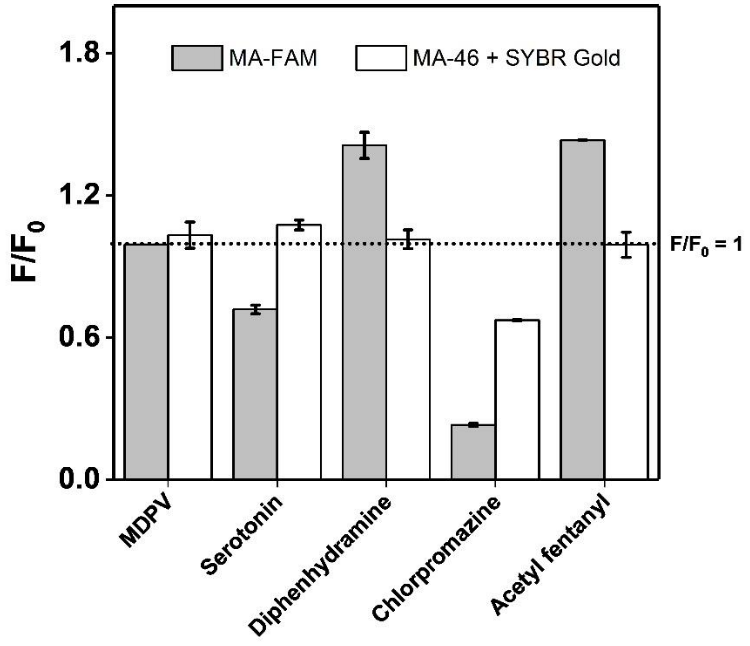

Figure S14. The effect of ligands on the fluorescence of the reporters used in the exonuclease-based profiling and strand-displacement assays. F/F_0 represents the ratio of the fluorescence of 50 nM MA-FAM or 1× SYBR Gold incubated with 400 nM unmodified MA-46 in the absence (F_0) or presence (F) of the ligand. Error bars represent the standard deviation of three experiments.

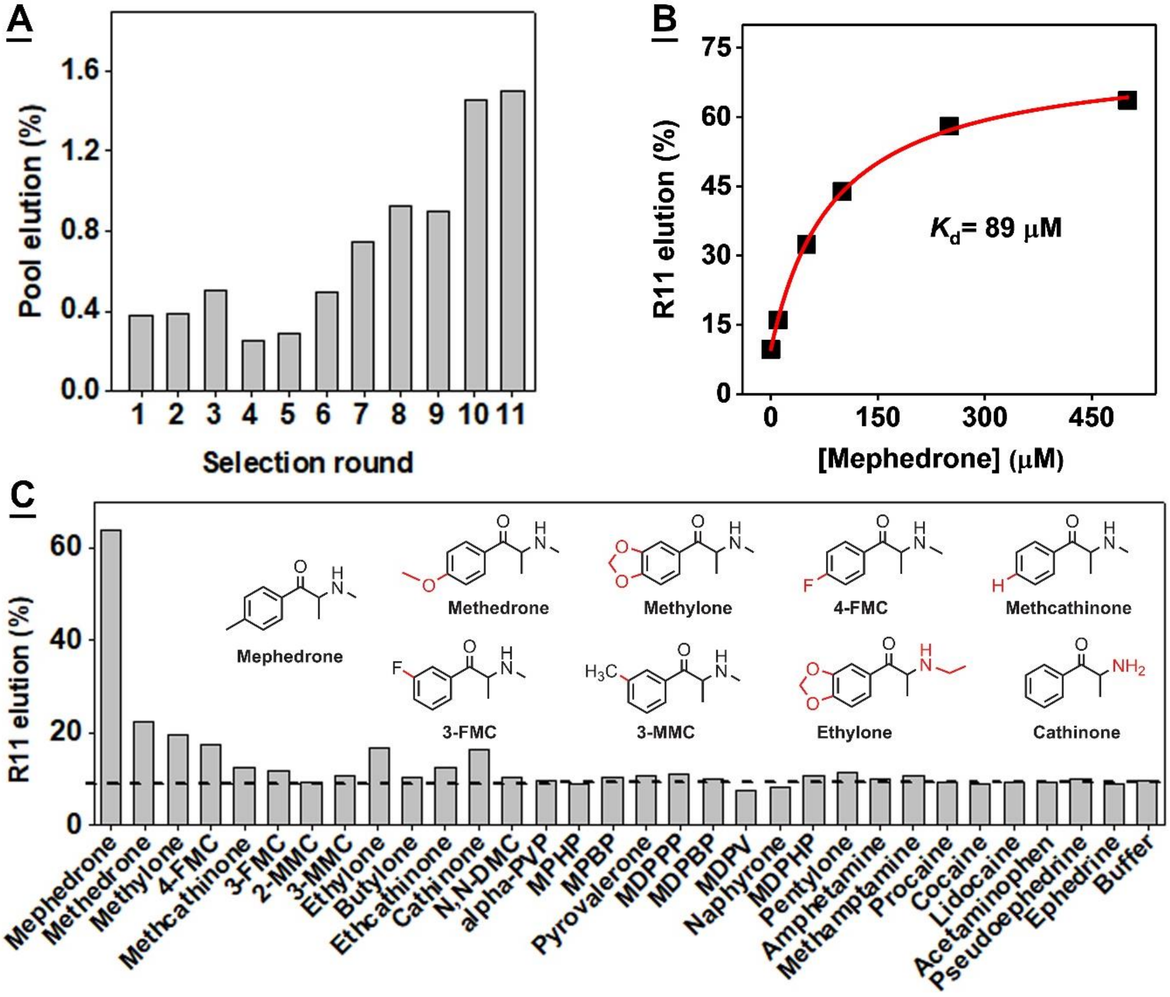

Figure S15. Isolation of a DNA aptamer that binds mephedrone via SELEX. (**A**) Mephedrone-induced pool elution determined after each round of SELEX. (**B**) Mephedrone-binding affinity of the final enriched pool (Round 11) as determined via the gel elution assay. (**C**) Percent of Round 11 pool eluted by mephedrone and 30 different counter-targets at a concentration of 500 μM. The dashed line represents the level of pool elution by the buffer alone (~10%). The structure of the compounds that yielded % elution values above those of buffer are provided, with moieties shaded in red accounting for the main difference between the ligand and mephedrone.

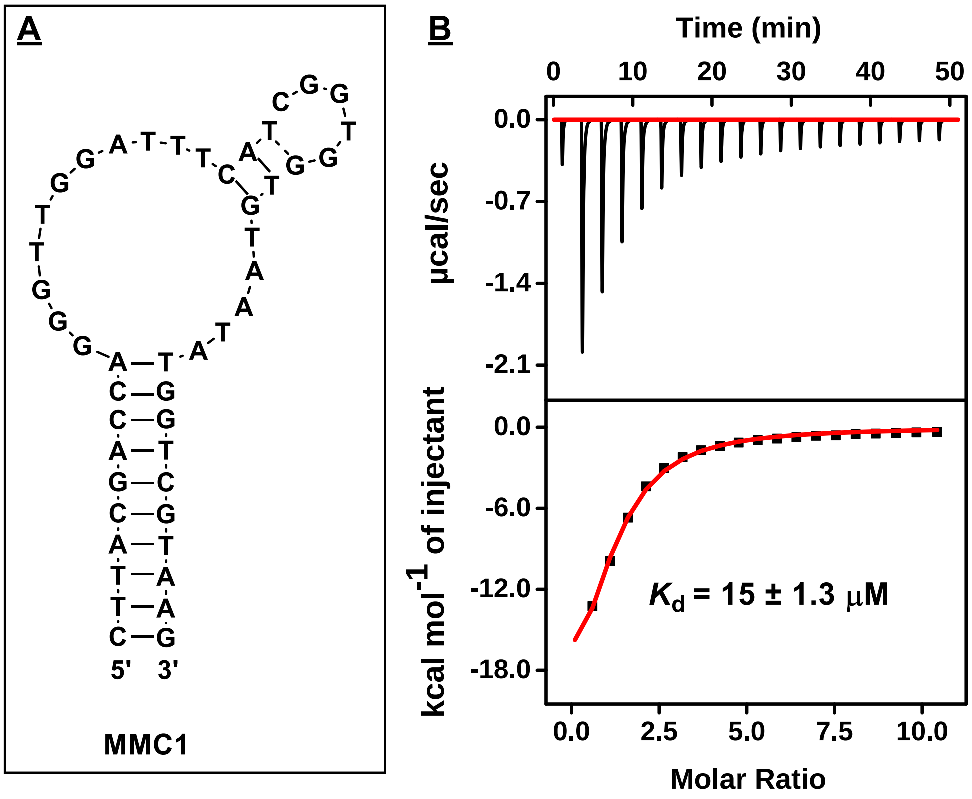

Figure S16. Characterization of the mephedrone-binding affinity of MMC1 using ITC. (**A**) Secondary structure of MMC1. (**B**) Binding affinity of its binding to mephedrone. The top panel presents raw data showing the heat generated from each titration of mephedrone to MMC1, while the bottom panel show the integrated heat of each titration after correcting for dilution heat of the titrant. ITC data were fitted with a one-site binding model.

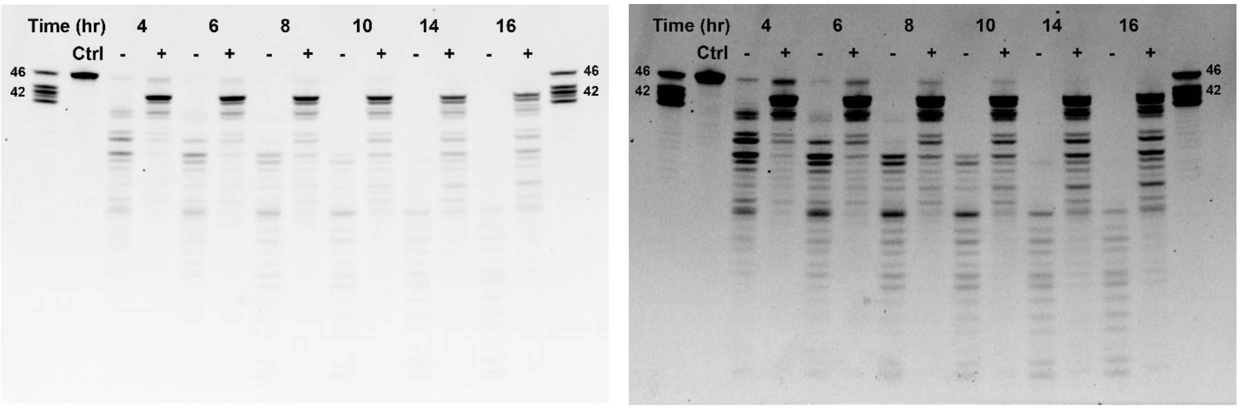

Figure S17. Time course digestion of MMC1 by T5 Exo in the absence (-) and presence (+) of 250 µM mephedrone analyzed by PAGE. Both images show the same gel, but at different levels of brightness to clearly show all products. Buffer: 10 mM Tris-HCl (pH 7.4), 20 mM NaCl, 0.5 mM $MgCl_2$.

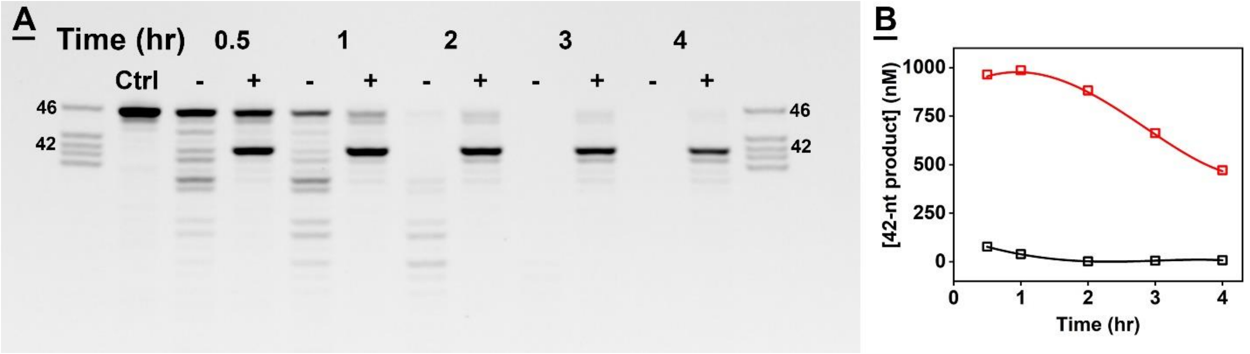

Figure S18. Digestion of MMC1 by the exonuclease mixture. (**A**) Time course digestion of MMC1 by T5 Exo and Exo I in the absence (-) and presence (+) of 250 μM mephedrone analyzed by PAGE. (**B**) Concentration of the 42-nt major product over time in the absence (black) and presence (red) of target. DNA concentrations were calculated relative to their respective ladder band.

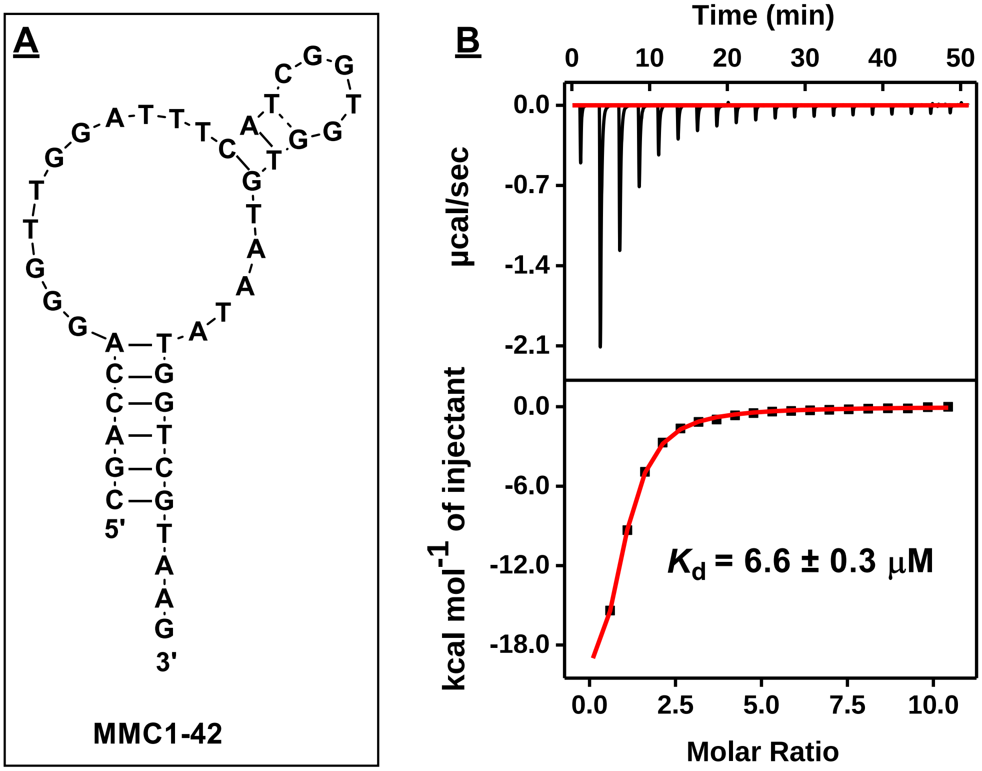

Figure S19. Characterization of the mephedrone-binding affinity of MMC1-42 using ITC. (**A**) Secondary structure of MMC1-42. (**B**) characterization of its binding affinity to mephedrone. The top panel presents raw data showing the heat generated from each titration of mephedrone to the aptamer, while the bottom panel show the integrated heat of each titration after correcting for dilution heat of the titrant. ITC data were fitted with a one-site binding model.

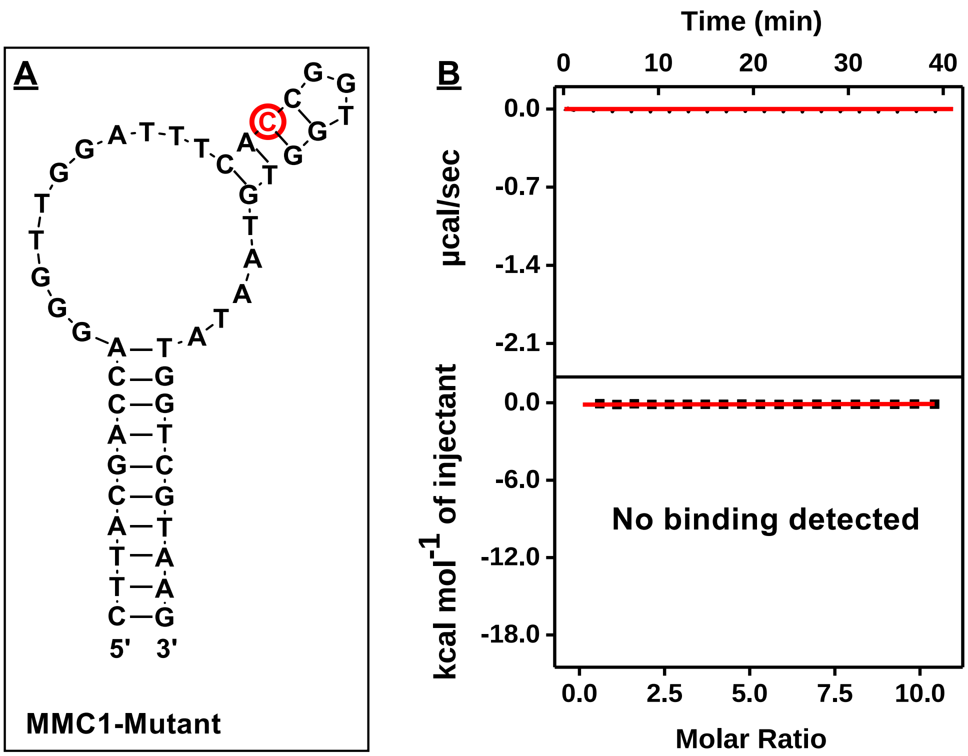

Figure S20. Characterization of the mephedrone-binding affinity of MMC1-Mutant using ITC. (**A**) Secondary structure of MMC1-Mutant. (**B**) characterization of its binding affinity to mephedrone. The top panel presents raw data showing the heat generated from each titration of mephedrone to the aptamer, while bottom panel shows the integrated heat of each titration after correcting for dilution heat of the titrant. ITC data were fitted with a one-site binding model.

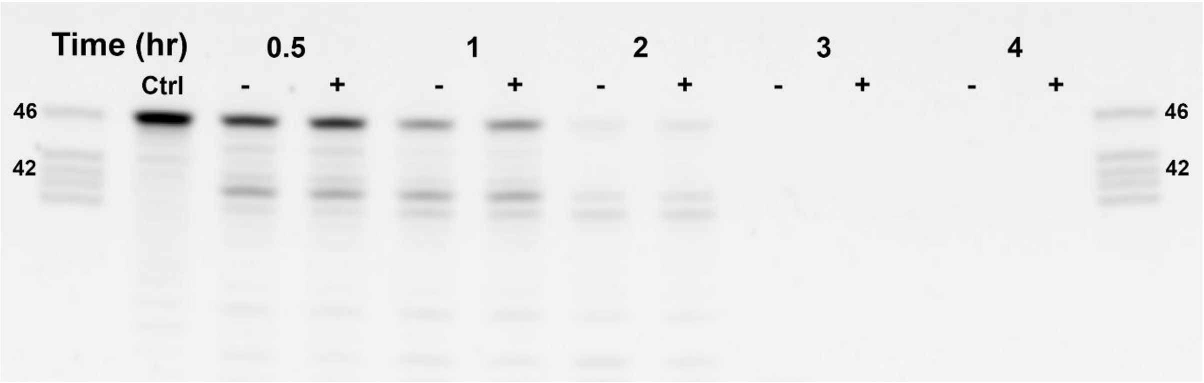

Figure S21. Time course digestion of MMC1-Mutant by T5 Exo and Exo I in the absence (-) or presence (+) of 250 µM mephedrone analyzed by PAGE.

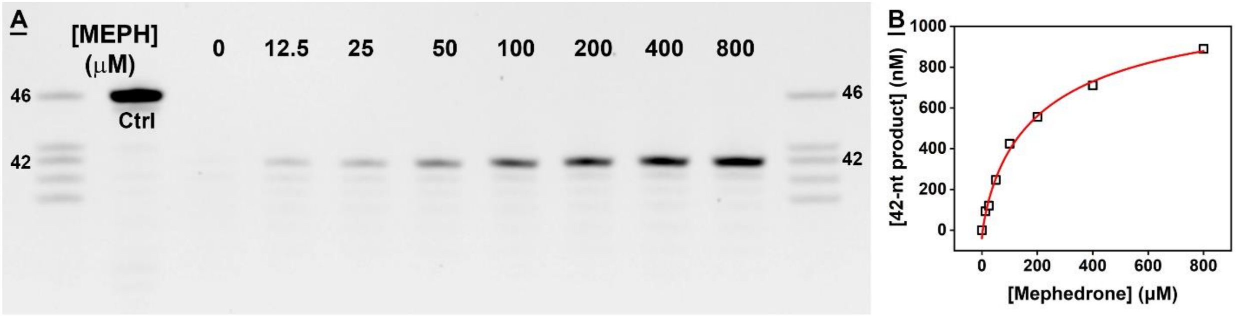

Figure S22. Target-concentration dependence of exonuclease inhibition for MMC1. (**A**) Digestion of MMC1 by T5 Exo and Exo I in the absence or presence of various concentrations of mephedrone after 3 hr analyzed by PAGE. (**B**) Concentration of the 42-nt major product as a function of the concentration of mephedrone.

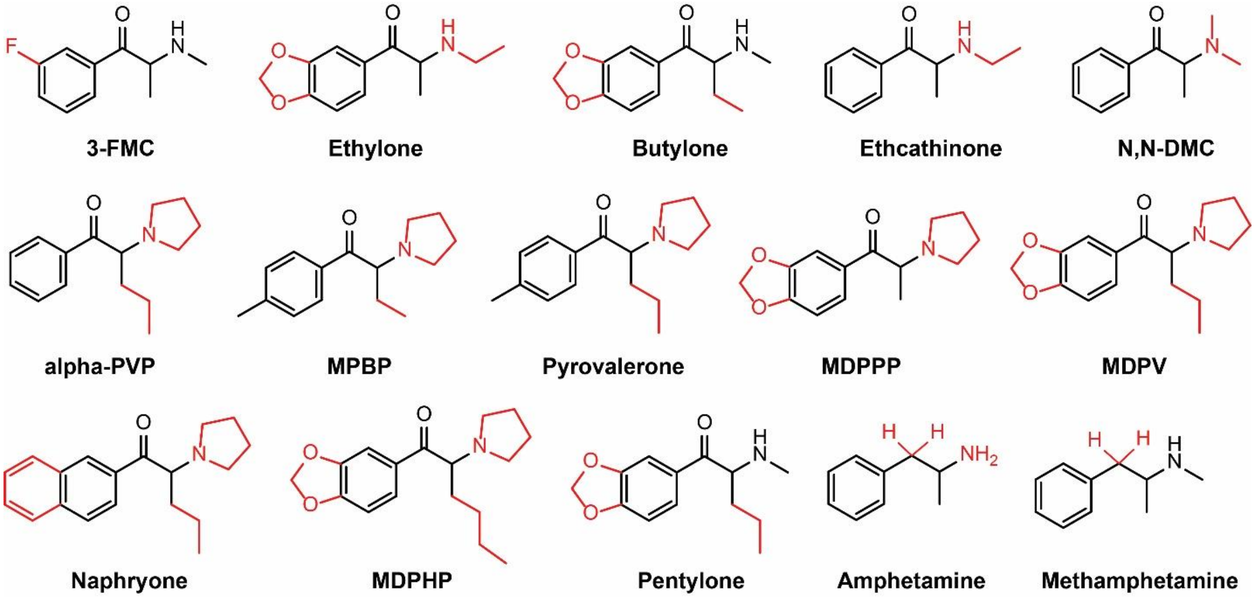

Figure S23. The chemical structures of other ligands with structural similarity tested in the exonuclease-based profiling assay with MMC1. Red-shaded portions are the main moieties that differ from mephedrone.

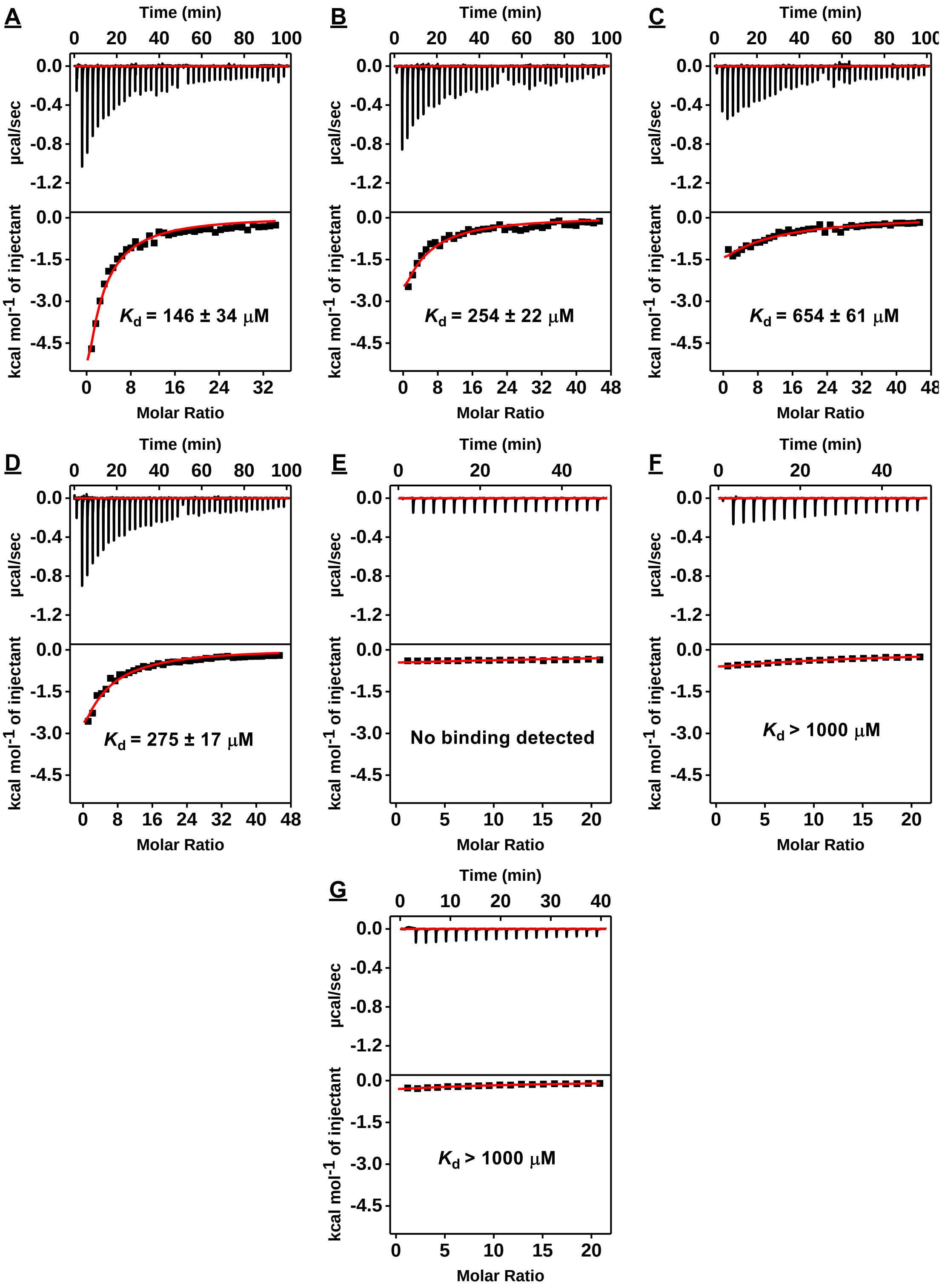

Figure S24. Characterization of the binding affinity of MMC1 to (**A**) methedrone, (**B**) ethylone, (**C**) methcathinone, and (**D**) methylone, (**E**) 2-MMC, (**F**) 3-MMC, (**G**) 4-FMC. Top panels present raw data showing the heat generated from each titration of ligand to the aptamer, while bottom panels show the integrated heat of each titration after correcting for dilution heat of the titrant. ITC data were fitted with a one-site binding model.

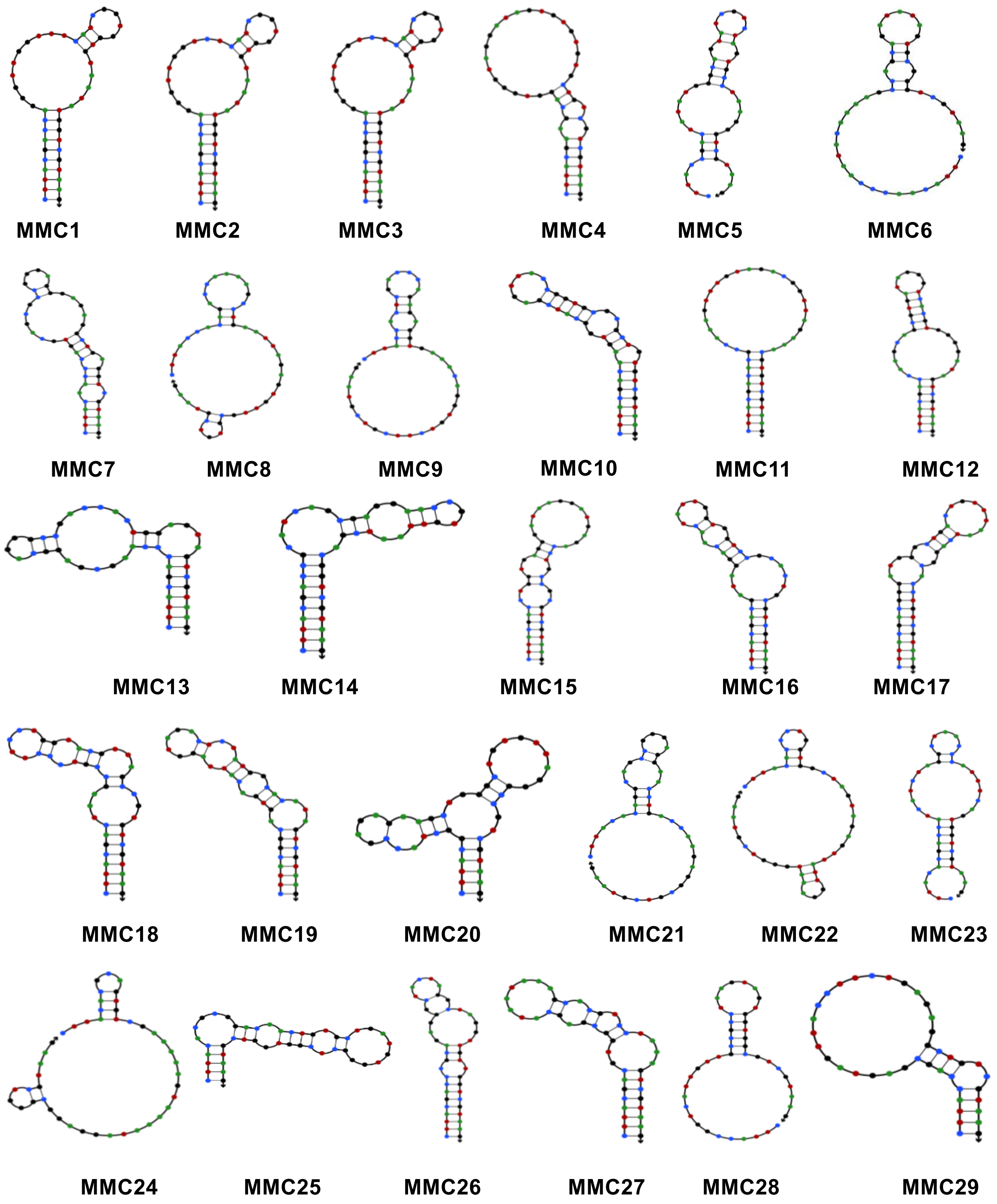

Figure S25. NUPACK-predicted secondary structures of MMC1-29. Green, red, black, and blue circles respectively represent A, T, G, and C. 3' ends are denoted by arrows.

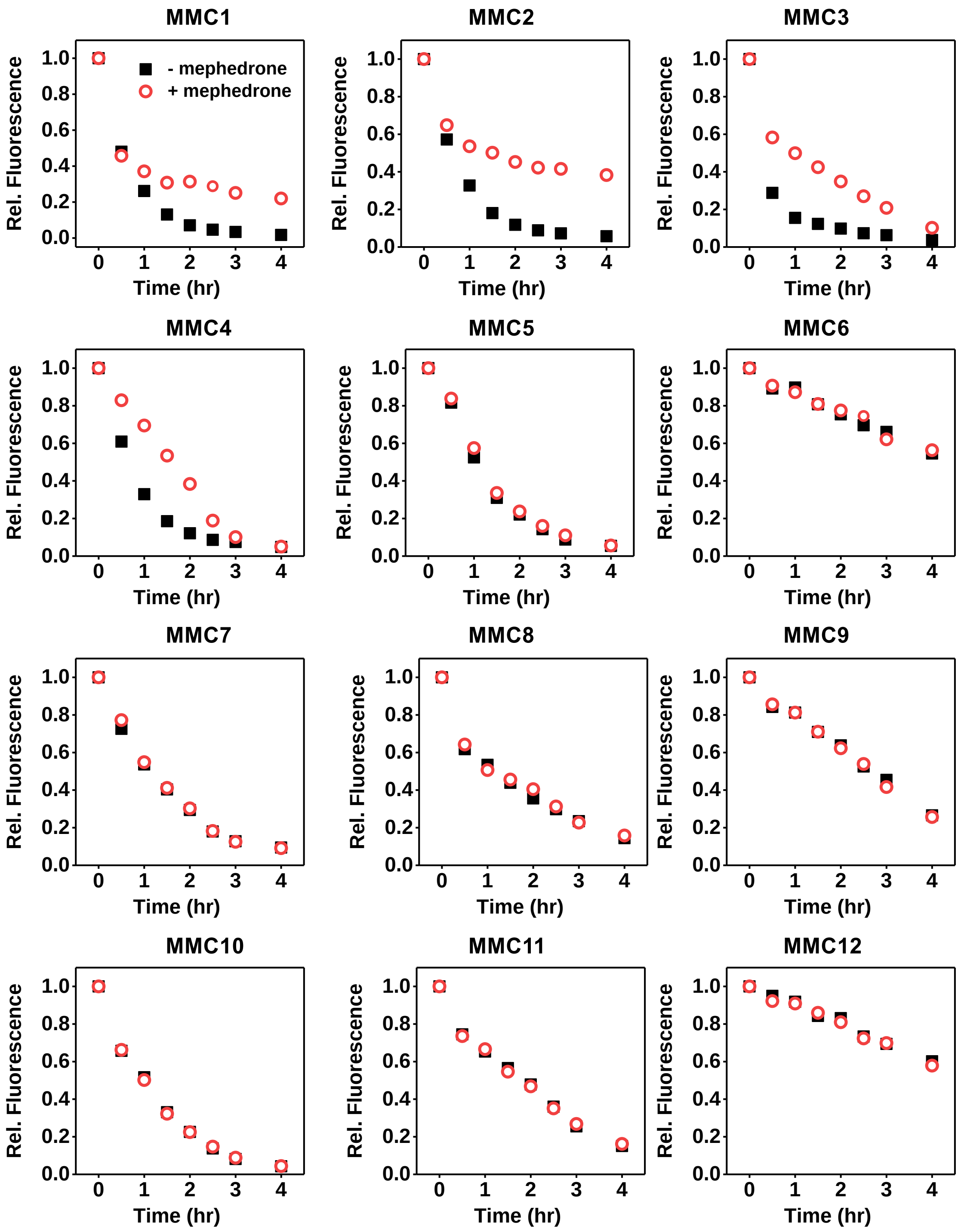

Figure S26. Using the exonuclease-based profiling assay to identify aptamer sequences that bind to mephedrone. Time course digestion plots of MMC1-12 are presented as relative fluorescence as a function of time. Solid black squares represent the fluorescence of samples with no target while open red circles represent the fluorescence of samples with 250 μM mephedrone.

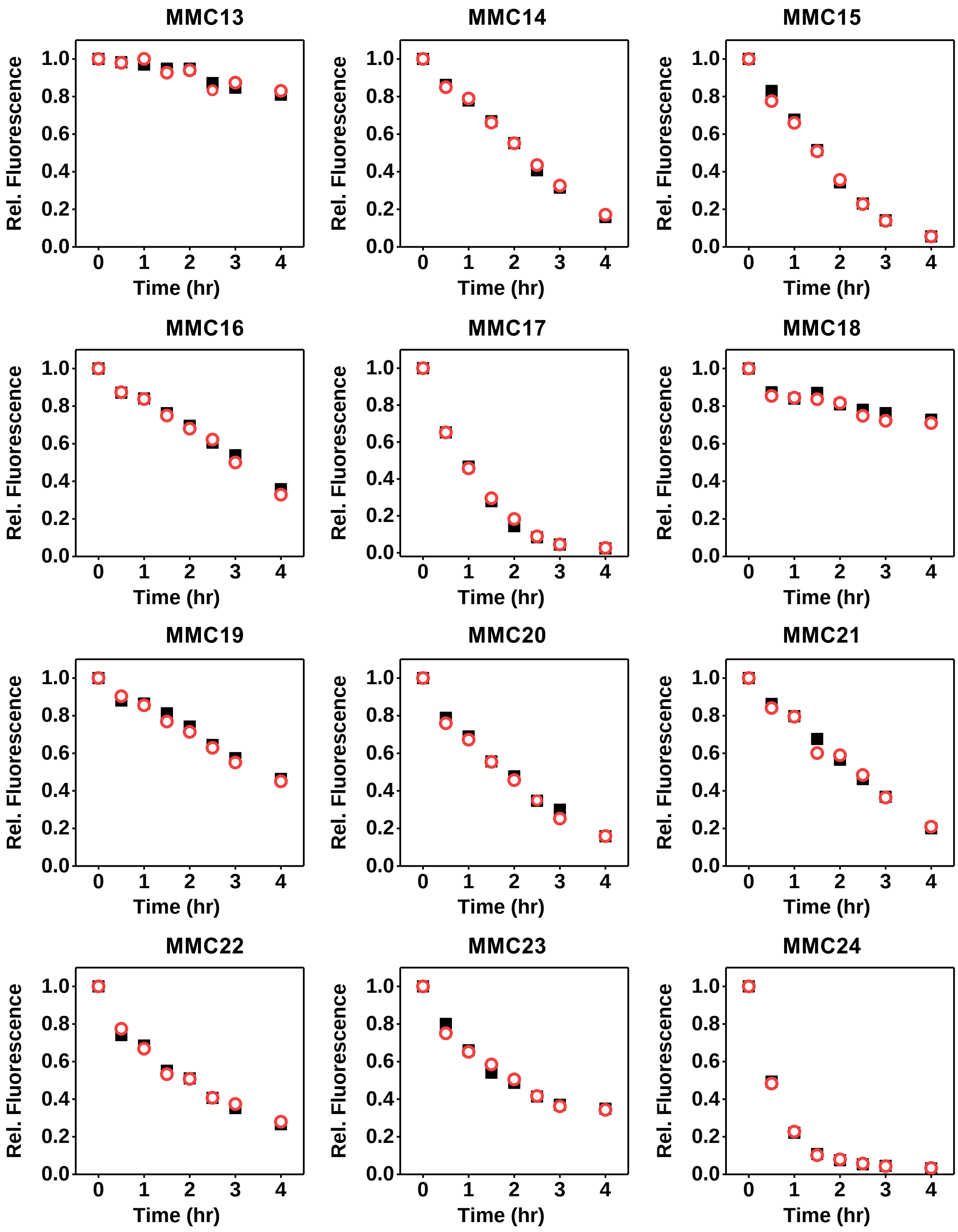

Figure S27. Using the exonuclease-based profiling assay to identify aptamer sequences that bind to mephedrone. Time course digestion plots of MMC13-24 are presented as relative fluorescence as a function of time. Solid black squares represent the fluorescence of samples with no target while open red circles represent the fluorescence of samples with 250 μM mephedrone.

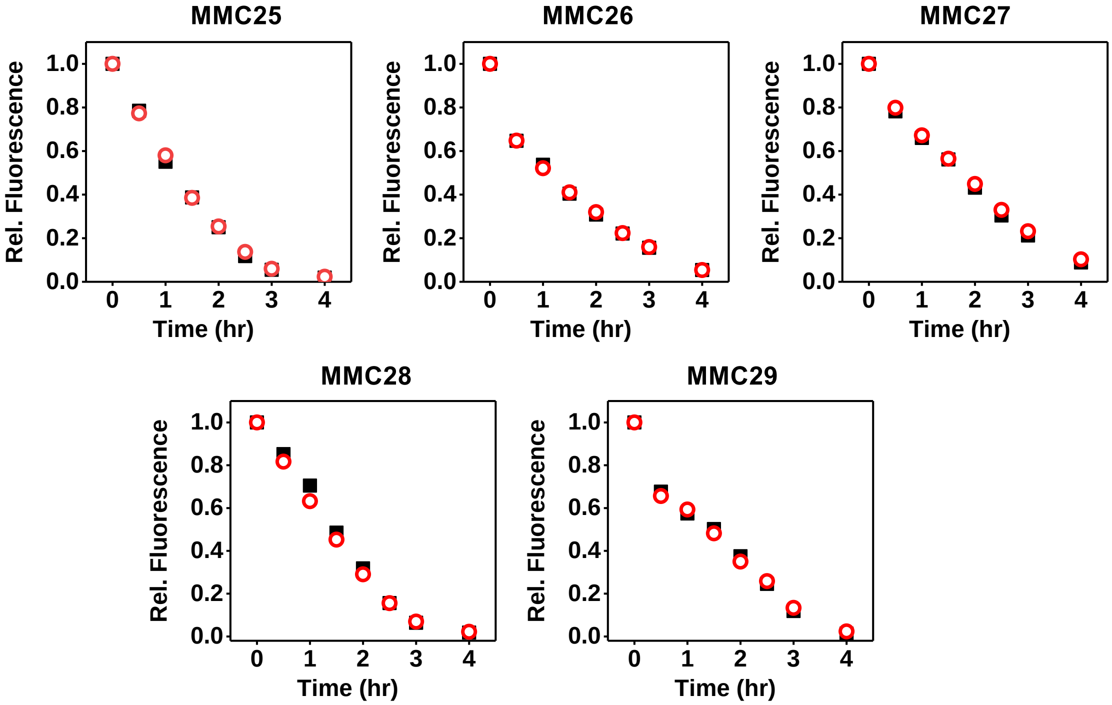

Figure S28. Using the exonuclease-based profiling assay to identify aptamer sequences that bind to mephedrone. Time course digestion plots of MMC25-29 are presented as relative fluorescence as a function of time. Solid black squares represent the fluorescence of samples with no target while open red circles represent the fluorescence of samples with 250 µM mephedrone.

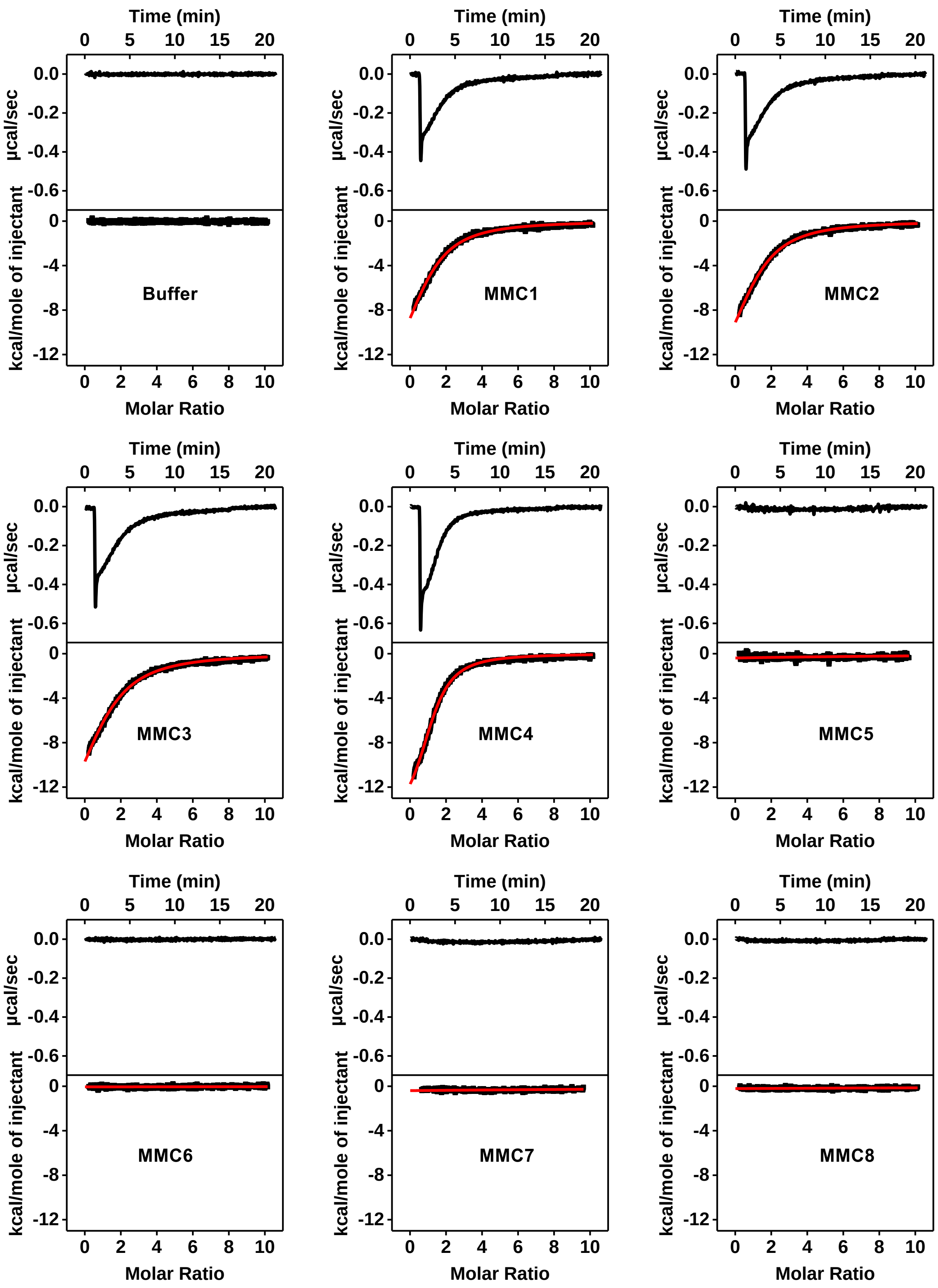

Figure S29. Characterization of the binding affinity of MMC1-8 to mephedrone using continuous-injection ITC. Top panels present raw data showing the heat generated over time as the ligand is titrated to the aptamer, while bottom panels show the integrated heat of each titration after correcting for dilution heat of the titrant.

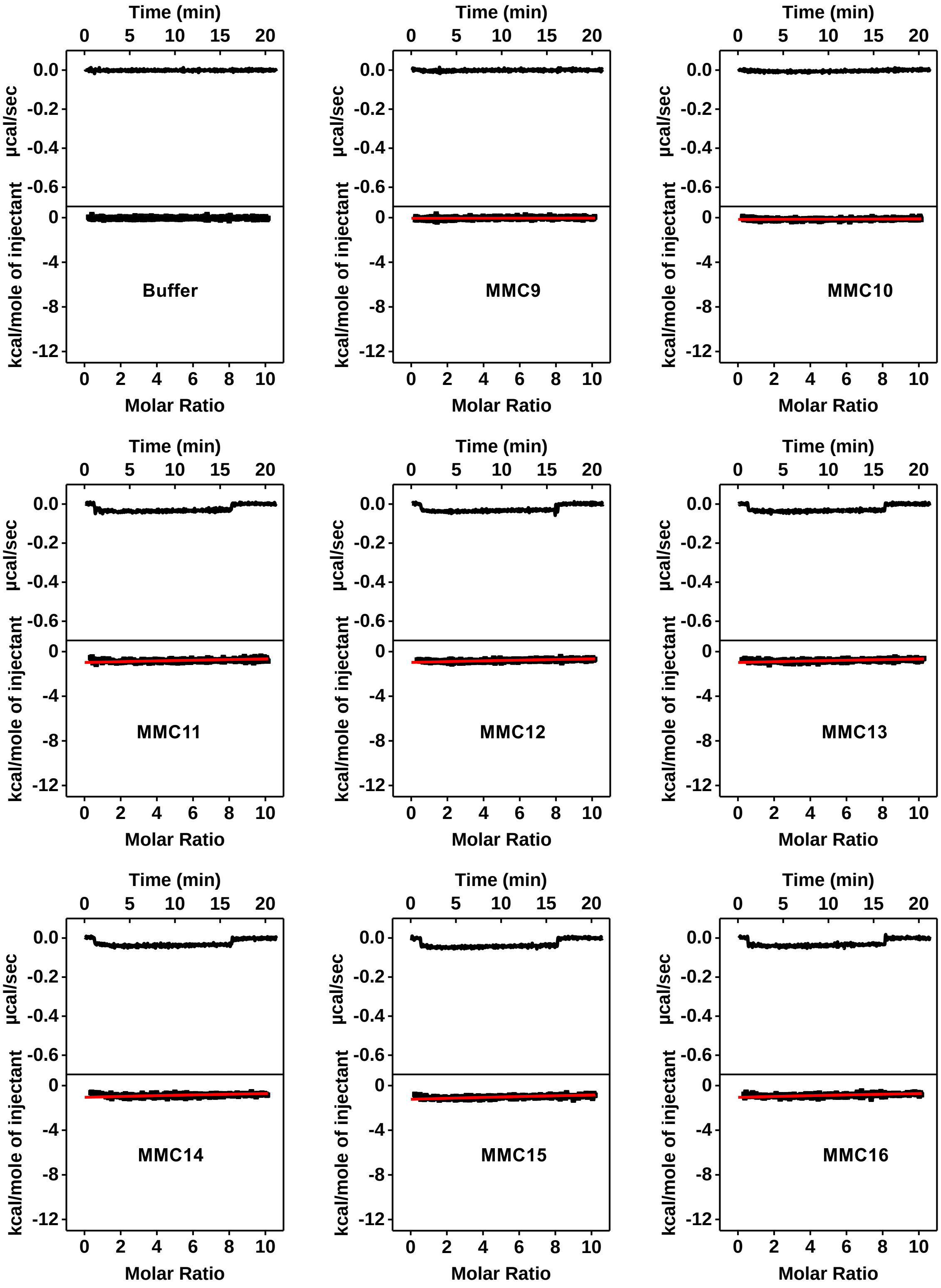

Figure S30. Characterization of the binding affinity of MMC9-16 to mephedrone using continuous-injection ITC. Top panels present raw data showing the heat generated over time as the ligand is titrated to the aptamer, while bottom panels show the integrated heat of each titration after correcting for dilution heat of the titrant.

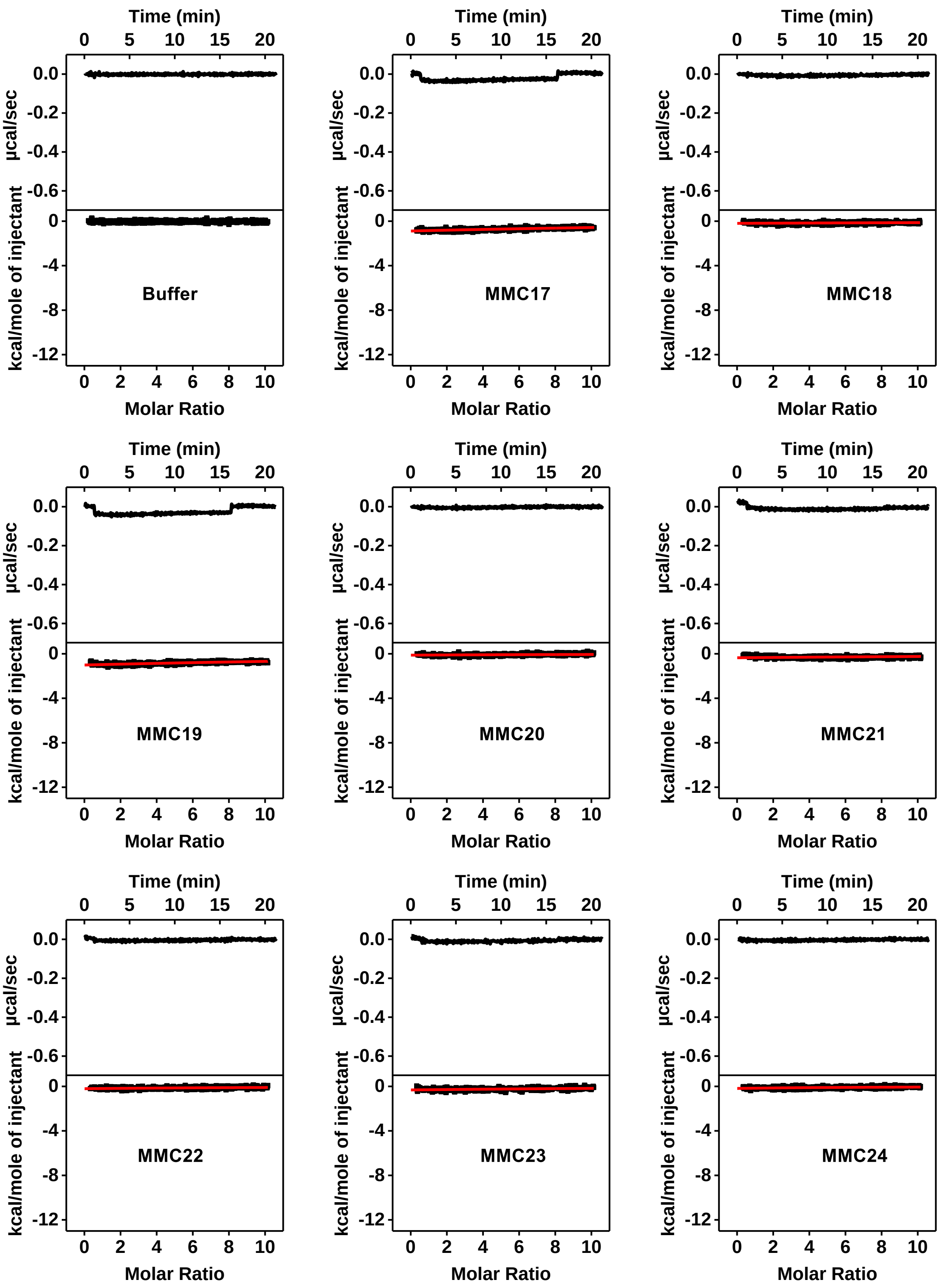

Figure S31. Characterization of the binding affinity of MMC17-24 to mephedrone using continuous-injection ITC. Top panels present raw data showing the heat generated over time as the ligand is titrated to the aptamer, while bottom panels show the integrated heat of each titration after correcting for dilution heat of the titrant.

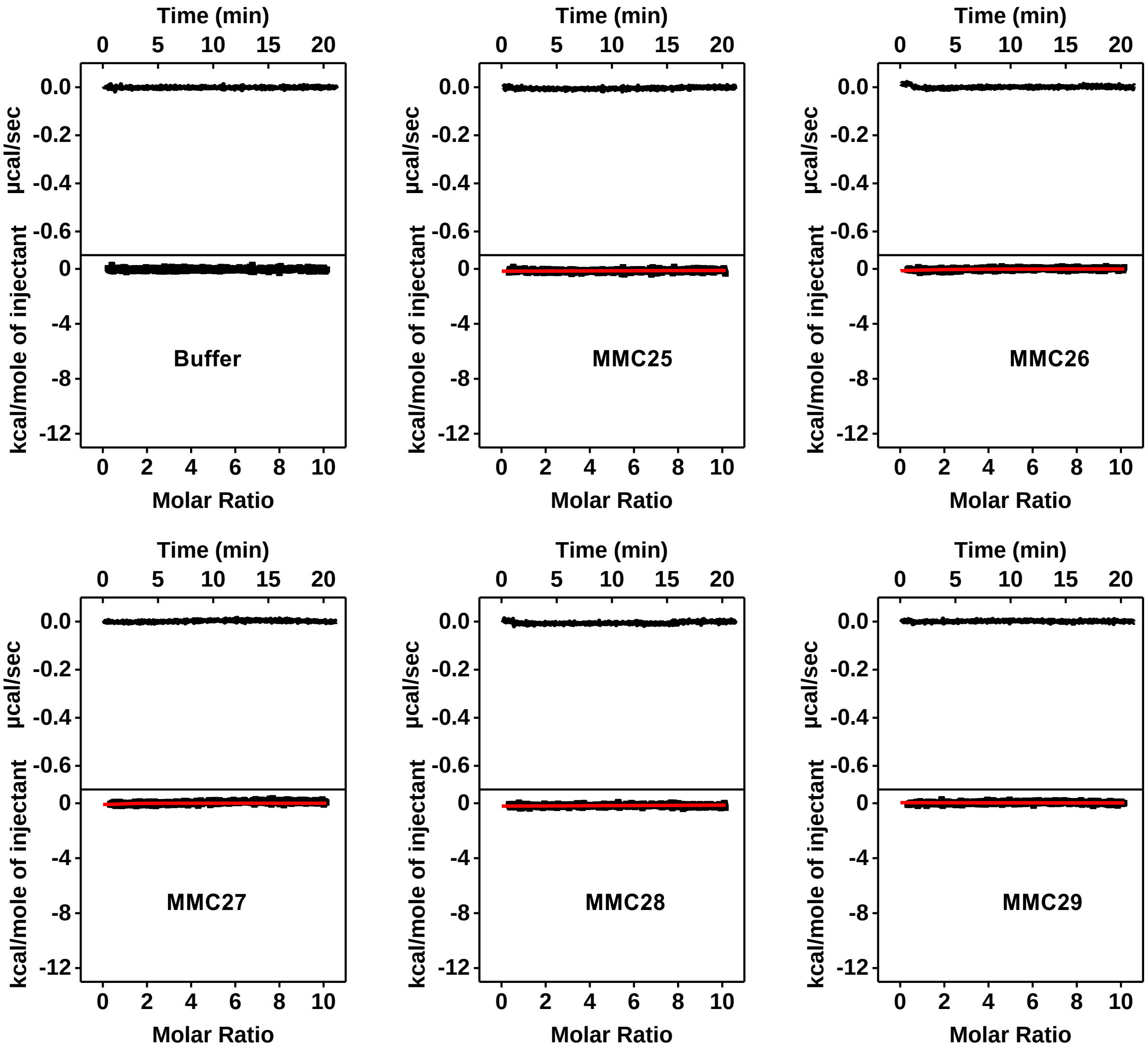

Figure S32. Characterization of the binding affinity of MMC25-29 to mephedrone using continuous-injection ITC. Top panels present raw data showing the heat generated over time as the ligand is titrated to the aptamer, while bottom panels show the integrated heat of each titration after correcting for dilution heat of the titrant.

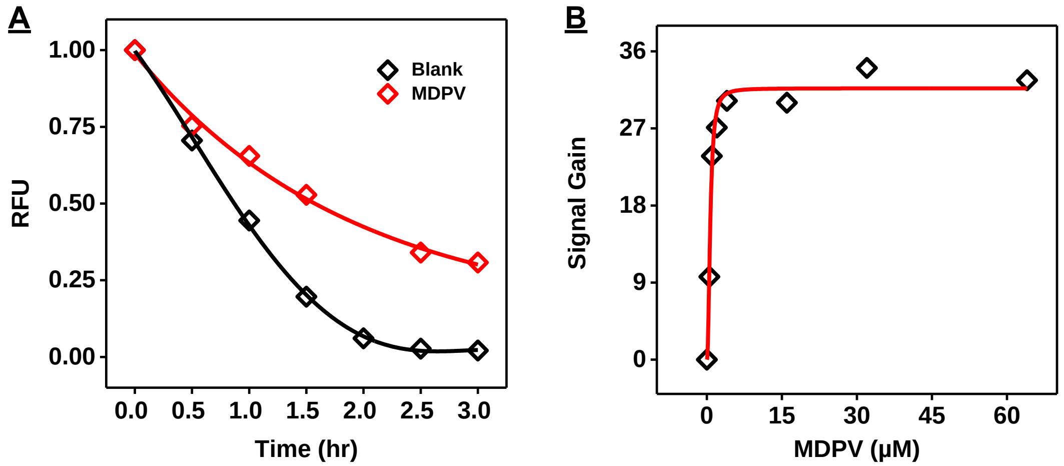

Figure S33. Digestion of SCA2.1 by a mixture of T5 Exo and Exo I. (**A**) Fluorescence time-course of SCA2.1 digestion in the absence (black) and presence of 50 μM MDPV (red). (**B**) Relationship between MDPV concentration and inhibition of enzymatic digestion of SCA2.1. SCA2.1 was digested in the presence of varying concentrations of MDPV (0, 1, 2, 4, 16, 32, or 64 μM), and signal gains of 3-hr digestion samples (F_1–F_0/F_0) are plotted against their corresponding MDPV concentration.

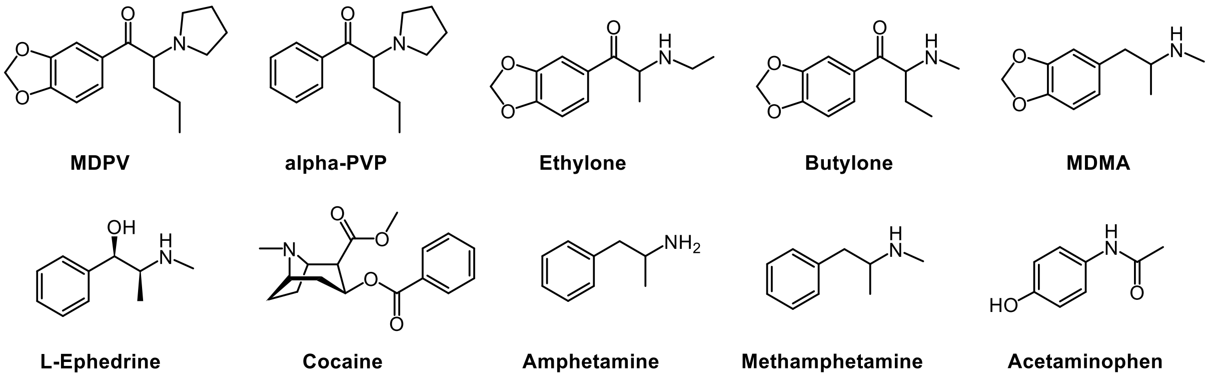

Figure S34. The chemical structures of the ligands tested in the exonuclease-based profiling assay with SCA2.1

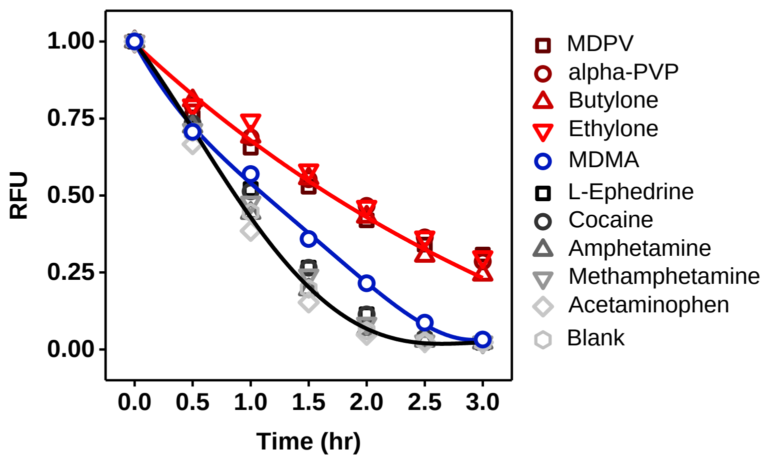

Figure S35. Fluorescence time-course of ligand binding profile of SCA2.1 as determined using the exonuclease-based profiling assay. The red symbols represent MDPV, alpha-PVP, butylone, and ethylone; the blue symbols represent MDMA; and the black and gray symbols represent L-ephedrine, cocaine, amphetamine, methamphetamine, and acetaminophen. The black line represents the fluorescence trend for the blank.

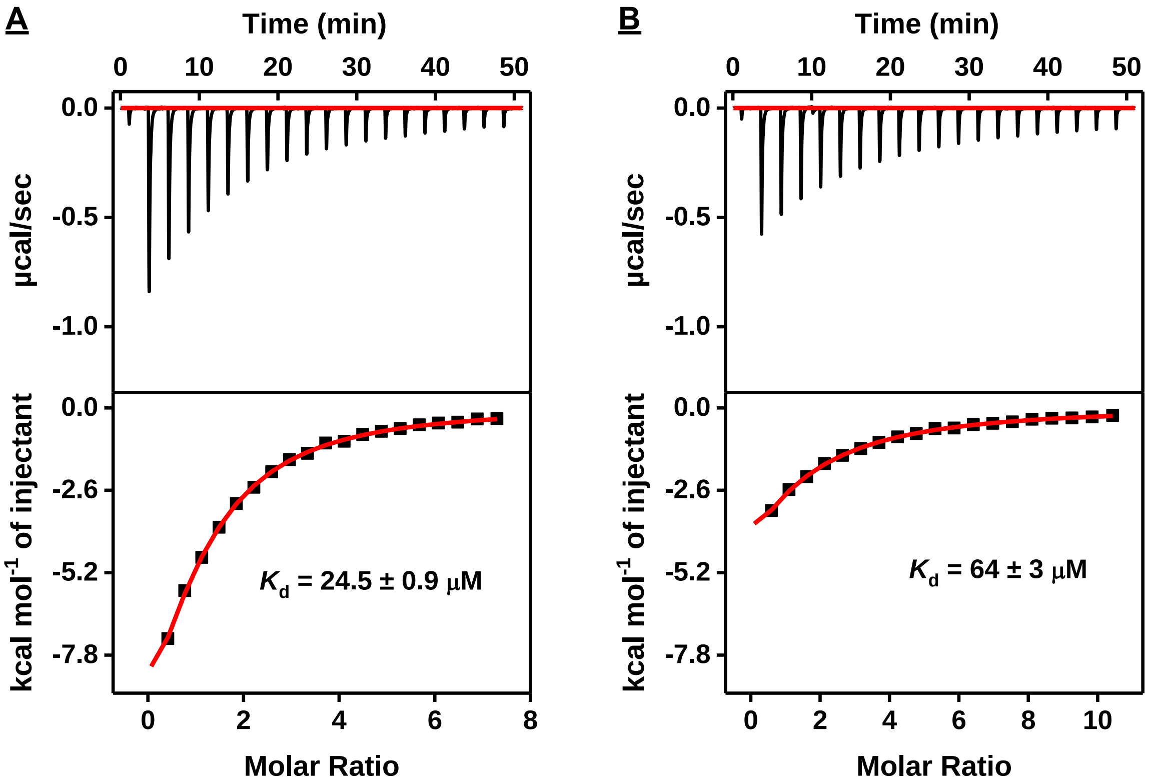

Figure S36. Binding affinity of (**A**) MDMA and (**B**) L-ephedrine for SCA2.1 determined using ITC. Top panels represent heat released per titration of ligand over time while bottom panels represent total heat values plotted against the molar ratio of ligand to aptamer. The isotherms were fit with a one-site binding model.

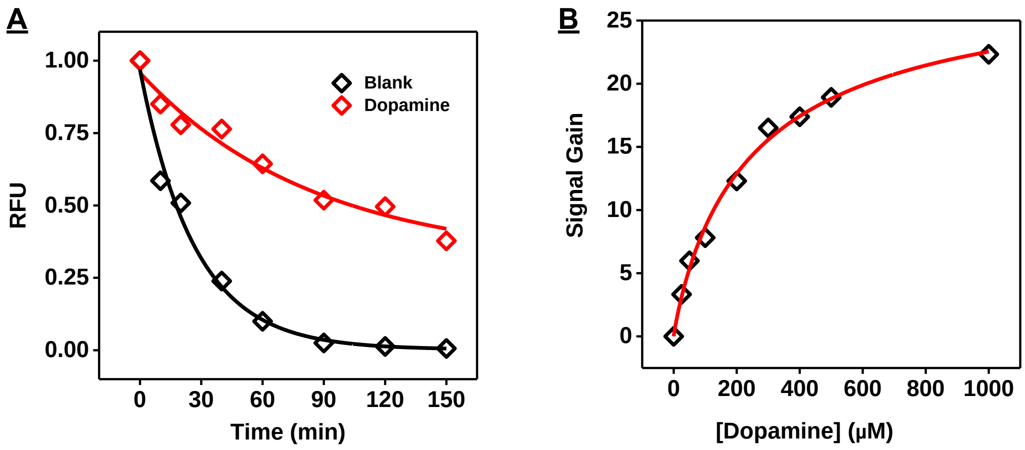

Figure S37. Digestion of dopamine-binding aptamer by a mixture of T5 Exo and Exo I. (**A**) Fluorescence time-course of digestion in the absence (black) and presence of 300 μM dopamine (red). (**B**) Relationship between ligand concentration and inhibition of enzymatic digestion. The aptamer was digested in the presence of varying concentrations of dopamine (0, 25, 50, 100, 200, 300, 400, 500, or 1,000 μM). Signal gains of 2.5-hr digestion samples (F_1–F_0/F_0) are plotted against their corresponding dopamine concentration.

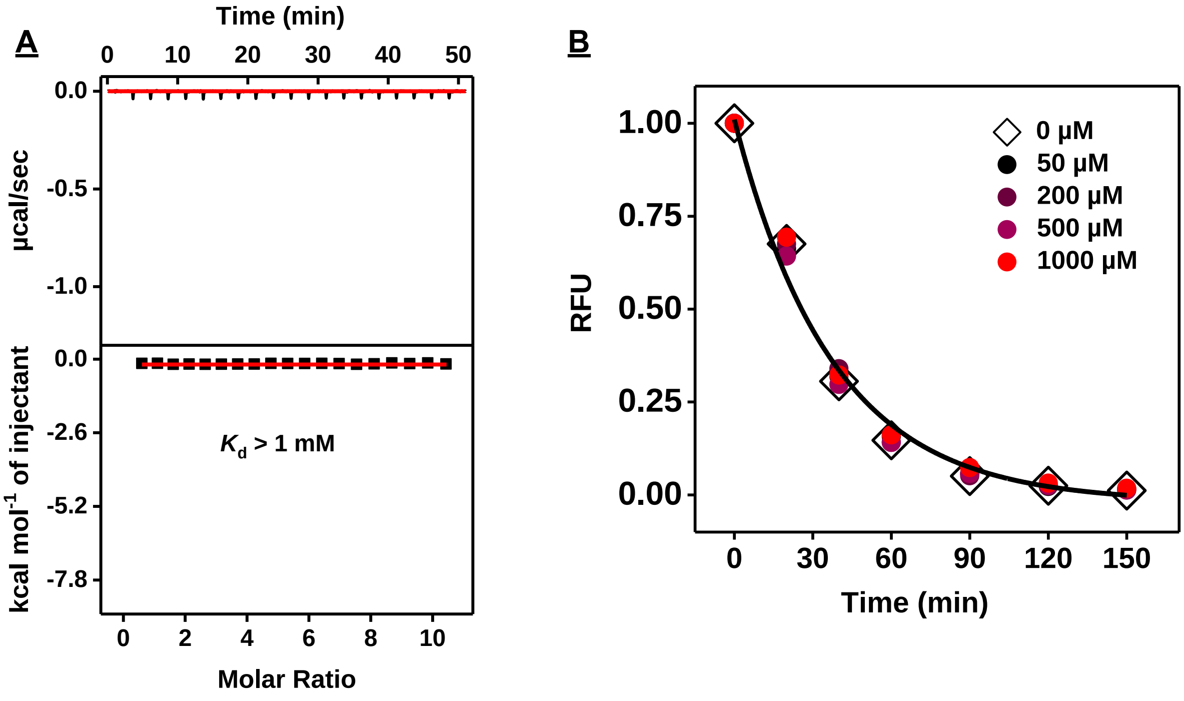

Figure S38. Interaction between MMC1 and dopamine. (**A**) Binding affinity of MMC1 to dopamine as determined by ITC. (**B**) Digestion of MMC1 by T5 Exo and Exo I in the presence of varying concentrations of dopamine.

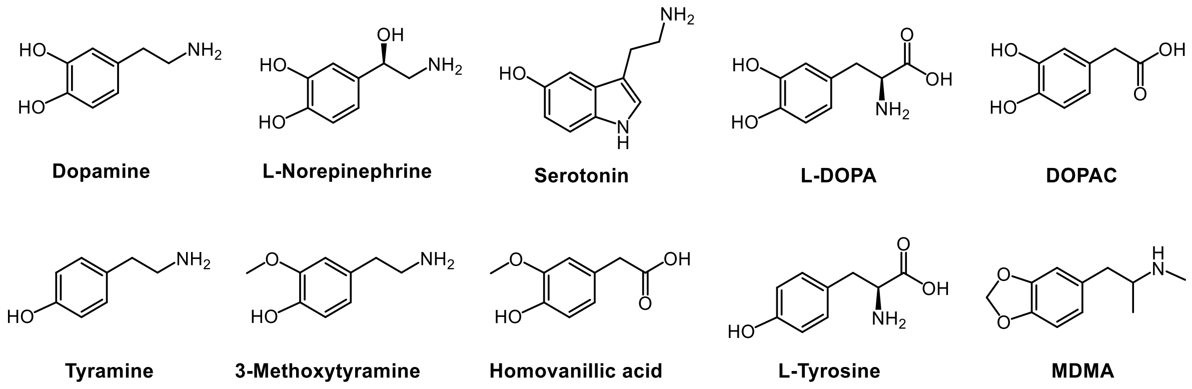

Figure S39. The chemical structures of the ligands tested in the exonuclease-based profiling assay with the dopamine-binding aptamer.

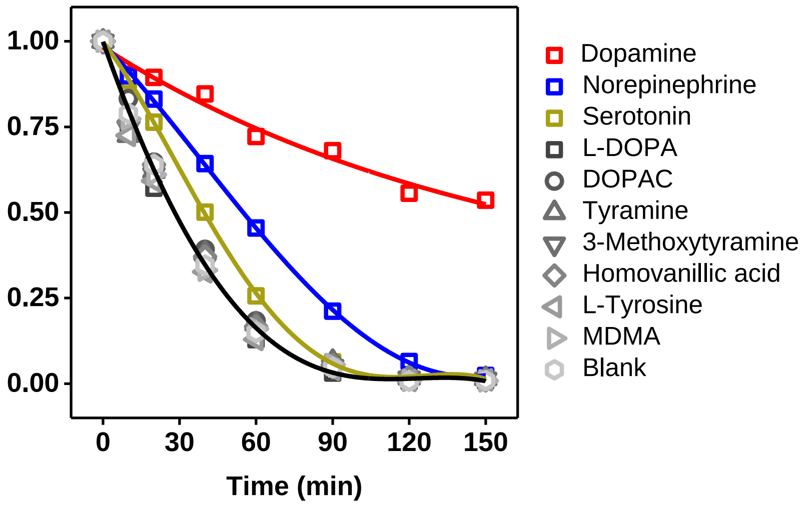

Figure S40. Fluorescence time-course of ligand binding profile of the dopamine-binding aptamer determined using the exonuclease-based profiling assay. Red, blue, and yellow symbols respectively represent dopamine, norepinephrine, and serotonin; black and gray symbols represent L-DOPA, DOPAC, tyramine, 3-methoxytyramine, homovanillic acid, L-tyrosine, and MDMA. The black line represents the fluorescence trend for the blank.
